# Historical data reveal extirpation of foundation species and kelp forest community deborealization in a coastal hotspot

**DOI:** 10.1002/eap.70223

**Published:** 2026-04-06

**Authors:** Brian Timmer, Luba Y. Reshitnyk, Christopher J. Neufeld, Julia K. Baum

**Affiliations:** ^1^ Department of Biology University of Victoria Victoria British Columbia Canada; ^2^ The Kelp Rescue Initiative Bamfield Marine Sciences Centre Bamfield British Columbia Canada; ^3^ Hakai Institute Heriot Bay British Columbia Canada; ^4^ LGL Limited Sidney British Columbia Canada; ^5^ University of British Columbia Okanagan Kelowna British Columbia Canada

**Keywords:** climate change, community temperature index (CTI), deborealization, extirpation, historical ecology, kelp forest, macroalgae, shifting baselines, species range shifts, tropicalization

## Abstract

Climate change is restructuring ecological communities globally, yet the impacts are often underestimated or poorly resolved due to the lack of historical baselines. In temperate oceans, biologically diverse and socioeconomically important kelp forests are the marine ecosystem most threatened by climate change. However, long‐term historical baselines for kelp forests are lacking and the processes driving community‐level changes remain poorly resolved. Here, using recently discovered aerial imagery and subtidal quadrat data from 1972, we recreated historical baselines for kelps and associated benthic macroalgae in a global hotspot within the northern Salish Sea (British Columbia, Canada). We resurveyed the same sites in 2023 to quantify community shifts, showing that a half‐century ago, bull kelp (*Nereocystis luetkeana*) formed expansive kelp forests in the region (>550 ha), none of which remain today. Satellite time series of bull kelp show that the majority was lost between 1972 and 1984. These data increase baselines of bull kelp canopy extent in this area by more than 10‐fold. Changes to the benthic kelp forest assemblage were mainly driven by loss of the dominant kelp, *Saccharina latissima* (−78%), across all depths. Historically abundant species of red algae also decreased substantially (e.g., *Mazzaella splendens* [−98.5%] and *Plocamium pacificum*, [−62.1%]), largely above three meters depth. Applying the community temperature index (CTI) to this half‐century comparison, we show that CTI of the kelp forest community (+1.4°C; 95% CI: 0.43–2.37°C) had tracked increases of summer SST (+1.66°C; 95% CI: 1.20–2.13°C) more closely than winter SST (+0.65°C; 95% CI: 0.46–0.84°C), indicating that temperatures during the hottest summer months are likely driving community shifts. The abundance of cold‐affinity species decreased more than warm‐affinity species abundance had increased, indicating that the subtidal kelp forest community was predominantly restructured by deborealization, rather than tropicalization. Community deborealization may be prevalent in temperate hotspots that are disjunct from areas with similar climatology, creating colonization barriers for warm‐affinity species. Our study underscores the importance of historical data for understanding the true magnitude of climate change impacts and suggests that deborealization of temperate kelp forest communities may be more common than has previously been recognized.

## INTRODUCTION

Anthropogenic climate change is warming oceans through both multi‐decadal temperature increases and punctuated marine heatwaves (Doney et al., 2012; Oliver et al., 2018), causing marine species to either move, adapt, or face extirpation (Harley et al., 2012; Parmesan & Yohe, 2003; Perry et al., 2005). At local and regional scales, microclimates can create hotspots in which extreme temperatures accelerate climate impacts on marine species (Hobday & Pecl, 2013; Pecl et al., 2014), causing marine communities to be restructured faster than regional averages (Burrows et al., 2019; Pinsky et al., 2013; Woodson et al., 2019). These hotspots may also be separated from regions of similar climatology by large distances, isolating them from potential source populations of warm‐adapted species, especially those with low dispersal potential (Harley et al., 2012). Understanding climate‐driven community shifts in these current and historical hotspots can give managers and policymakers insight into how future climates could restructure marine communities in slower warming regions (Blois et al., 2013; Pecl et al., 2014, 2017), allowing practical, climate‐smart decision‐making around marine resource management (Frazão Santos et al., 2024; Lawler et al., 2010).

Accurate historical baselines are crucial for detecting long‐term climate impacts in marine ecosystems (McClenachan et al., 2012). Historical baselines are generally used to contextualize modern changes in an ecosystem versus a pre‐disturbance state, making the temporal scales at which historical baselines are set important as well (Foster, 2000). Technological advances and increased monitoring efforts in recent decades have increased our knowledge of more recent changes in marine ecosystems, yet climate change has been ongoing for over a century, and the use of baselines set in recent decades may obscure longer term climate impacts and result in “shifting baseline syndrome” where perceptions of the natural environment shift over time (Pauly, 1995). Unfortunately, long‐term ecological time‐series data are generally rare, making historical baselines, and thus, long‐term changes, difficult to identify (McClenachan et al., 2024; Soga & Gaston, 2018). The scarcity of long‐term time series makes the use of historical data for setting ecological baselines, although often imperfect, a powerful way to measure ecosystem changes which would otherwise have been lost to time. Historical changes detected from these baselines can also inform marine managers of future changes that may soon occur in similar regions or systems (Pecl et al., 2014).

**FIGURE 1:**
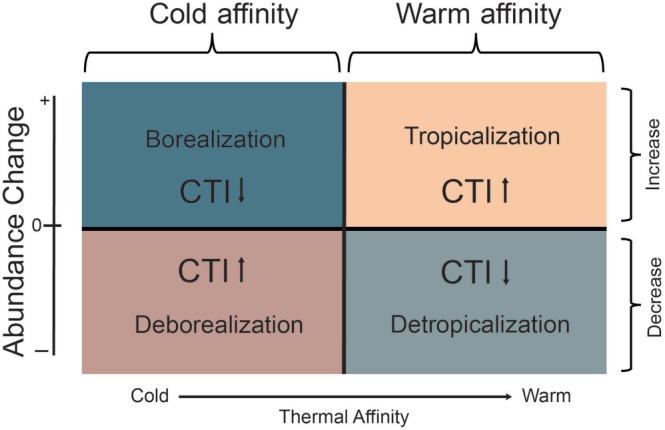
The four processes that contribute to shifts in community temperature index according to change in abundance and thermal affinity (adapted from McLean et al., 2021).

Historical community shifts can be characterized by tracking changes in the community temperature index (CTI), a measure of the mean thermal affinity of species within a community (Devictor et al., 2008, 2012). As oceans warm, the relative abundance of warm‐affinity species in a community increases, as does the CTI (Figure 1). There are two underlying processes that can increase CTI (McLean et al., 2021); either the total abundance of warm‐affinity species in a community can increase, known as tropicalization, or the total abundance of cold‐affinity species can decrease, known as deborealization (McLean et al., 2021). Both processes increase the relative abundance of warm‐affinity species in a community, while their inverse processes (detropicalization and borealization) decrease the relative abundance of warm‐affinity species (Figure 1), thus decreasing the CTI (McLean et al., 2021). While these processes are generally used to document range shifts related to climate change, they may also be correlated with anthropogenic introductions, species interactions, or other anthropogenic factors (habitat destruction, overharvest, etc.).

Recent marine heatwaves have been linked to the tropicalization of kelp forests at both population (i.e., genetically) and community levels (Coleman et al., 2020; de Azevedo et al., 2023) along gradients of warming between tropical and temperate climates. Related research has documented the impacts of tropicalized fish communities on habitat‐forming kelp forest canopies (Vergés et al., 2014; Zarco‐Perello et al., 2017). Yet, the vast majority of kelp forests exist along complex temperate coastlines in the northern hemisphere, often with low connectivity to tropical and subtropical waters (Starko et al., 2019; McHenry et al., 2025; Krumhansl et al. 2016), and long‐term community‐level shifts related to climate change are poorly understood for kelp forest communities in these regions.

Understanding climate change impacts on kelp forests is crucial because they form critical nearshore habitat, including nursery and feeding grounds for many pelagic and deep‐water fishes, and are also the base of nearshore food webs (Corliss et al., 2024; Kennedy et al., 2018; Shelton et al., 2014). Kelp forest communities are formed by diverse macroalgae taxa with highly variable morphologies and life histories (Druehl, 1970; Starko et al., 2019), but are generally dominated by kelps (Order Laminariales, but often including other large species in Class Phaeophyceae), which are the most productive group of macroalgae (Wernberg & Filbee‐Dexter, 2019). Their productivity and dominance lend kelps the status of “foundation species”—species that structure an ecosystem through functional roles like habitat provisioning or nutrient cycling (Dayton, 1972; Dayton et al., 1984)—within temperate nearshore ecosystems. Moreover, kelp forests have created longstanding cultural and socioeconomic benefits to Indigenous and coastal communities through both direct use of the kelps as well as the ecosystems they support (Eger, Marzinelli, et al., 2023; Turner, 1995; Dillehay et al., 2008). Kelp forests are also being investigated as a natural climate solution (McHenry et al., 2025). As such, there is growing recognition of the importance of kelp forests globally, and the increased need for proper management of kelp forest ecosystems through both restoration and conservation (Eger, Aguirre, et al., 2023), especially with kelp forests being listed by the Intergovernmental Panel on Climate Change as the temperate marine ecosystem most vulnerable to climate change (Bindoff et al., 2019). Yet, the ability to effectively manage kelp forest ecosystems is dependent on both understanding and mitigating stressors (Hollarsmith et al., 2022), meaning it is crucial for managers and decision‐makers to understand long‐term trends and drivers of change in kelp forest communities.

Among kelps and other macroalgae, the distribution of species that form floating surface‐canopies is relatively well documented historically because of their ecological importance (Dayton, 1985), ease of detection via remote sensing (Cavanaugh et al., 2021), and historical mapping as navigational hazards (Costa et al., 2020; Hollarsmith et al., 2024; Mora‐Soto et al., 2021) or for harvesting interests (Cameron, 1914). Satellite‐derived time series have been especially useful for monitoring kelps and have demonstrated close linkages between interannual variability of kelp extent and large‐scale oceanographic processes like the Pacific Decadal Oscillation (PDO) and El Niño/La Niña cycles (Cavanaugh et al., 2011; Cavanaugh et al., 2021; Gendall et al., 2025; Hamilton et al., 2020). Despite the valuable ecological insights gained from the use of satellite imagery, there are also numerous considerations related to environmental and sensor parameters, especially when using older satellite imagery, that can limit detections of historical kelp canopies (see Cavanaugh et al., 2021 for detailed overview), making alternative historical data crucial to developing accurate historical kelp baselines and understanding timelines for kelp losses (Costa et al., 2020; Hollarsmith et al., 2024).

In the northeast Pacific, bull kelp (*Nereocystis luetkeana*) is a surface‐canopy forming species that has declined substantially in many regions, generally due to either extreme heat events, overgrazing by sea urchins, or some combination of these two processes (Finger et al., 2021; Rogers‐Bennett & Catton, 2019; Starko et al., 2024). While both of these processes can result in surface‐canopy losses, their comparative effects on understory macroalgae are less understood. In regions where *Nereocystis* forests have been lost due to overgrazing by urchins, “urchin barrens” generally form an alternative stable state in which the vast majority of benthic macroalgae are also lost from the system and only coralline algae or small turf algae remain (Filbee‐Dexter & Scheibling, 2014; Rogers‐Bennett & Catton, 2019). Conversely, where *Nereocystis* forests have been lost due to warming (Starko et al., 2024), much less is known about changes in the benthic macroalgae of these kelp forest communities. With ocean warming expected to continue under climate change, it is crucial for managers and policymakers to understand the extent of climate change impacts on these benthic habitat‐forming species to guide decision‐making around their conservation and restoration (Hollarsmith et al., 2022).

Our objectives in this study were to leverage newly rediscovered historical data to uncover how climate change‐induced ocean warming has altered the extent of a critical marine foundation species, bull kelp (*N. luetkeana*), and the associated kelp forest community over the past half century in a temperate coastal hotspot (Hobday & Pecl, 2013). To do so, we reconducted ecological surveys in the Salish Sea—a semi‐enclosed body of water comprised of numerous waterways between British Columbia, Canada, and Washington State, USA—during the summer of 2023 using consistent methodologies at the same nearshore sites as those in historical surveys from the summer of 1972. We hypothesized that (**H1**) rapid warming in ocean hotspots would have produced temperatures decades ago that exceeded the thermal limits of habitat‐forming foundation species, resulting in large‐scale *Nereocystis* losses well before the documented effects of recent marine heatwaves. We also hypothesized that (**H2**) the CTI of the benthic kelp forest community in this coastal hotspot had increased in recent decades, largely due to restructuring via deborealization, rather than tropicalization, because warmer temperatures had reduced the abundance of cold‐affinity species and the isolation of this temperate microclimate had limited new warm‐affinity species from moving in. The investigation of these hypotheses highlights potential climate change related habitat shifts at both foundation species and community levels, serving as a window into the challenges coastal ecosystems have faced and will continue to face under climate change.

## METHODS

### Study region

The coast of British Columbia (BC), Canada, is over 25,000 km in length and highly complex, formed by a combination of glacial and tectonic forces that have created numerous microclimates along its shoreline (Thomson, 1981). Some of the most extreme microclimates in BC, both warm and cool, can be found in the Salish Sea, a semi‐enclosed body of water comprising numerous waterways between BC, Canada, and Washington State, USA. Some regions of the Salish Sea are strongly mixed by tidally driven exchange, creating cool and stable refugia for kelp forests (Mora‐Soto et al., 2024b; Pfister et al., 2017), while other regions have experienced continuous warming punctuated with intensifying marine heatwaves over the last half century (Bond et al., 2015; Chen et al., 2021; Di Lorenzo & Mantua, 2016; Masson & Cummins, 2007). Generally, extreme heat events such as marine heatwaves are more likely to occur during natural fluctuations within PDO and El Niño/La Niña cycles, yet the intensity and duration of these extreme heat events are also being amplified due to climate change (Athanase et al., 2024; Cai et al., 2021). Within warmer microclimates, marine heatwaves have greatly reduced surface‐canopy kelps in recent decades (Starko et al., 2024). The Strait of Georgia is the largest body of water in the Salish Sea, in part defined by a unique microclimate with relatively warmer temperatures than most other regions of the Salish Sea (Amos et al., 2015). The tidal range extends up to five meters above chart datum (Canadian Hydrographic Service, 2025), and during summer months, a strong thermocline is established that generally persists until late summer and fall storms mix the upper layers of water column (Thomson, 1981). Our study focuses on the western coastline of the Strait of Georgia between Miracle Beach (49.86009, −125.11131) to the southern end of Denman Island (49.49060, −124.70136), where the gradually sloping shores create expansive shelfs of shallow, nearshore habitat. The shorelines and waterways within this study are the traditional territories of multiple First Nations, and directly adjacent to the Treaty Lands of the K'ómoks First Nation. We acknowledge that members of these First Nations hold deep knowledge of the region dating back much farther than the timelines of the historical data used in our study, and that our results here are but one way of seeing/knowing in a world of many knowledge systems (Grimm et al., 2025; Reid et al., 2021).

### Environmental change

We used a combination of historical and modern oceanographic data to quantify spatial and temporal oceanographic trends within the study region and to understand those trends within the broader context of both regional and global climate change. First, to characterize the study region over the past half century, measurements of sea surface temperature (SST) and practical salinity units (PSUs) were taken from the Chrome Island lighthouse time series, a government run lighthouse station at the southern edge of Denman Island with daily SST and PSU measurements dating back to 1961. We used a series of generalized additive models (GAMs; mgcv package in “R”) to model SST and PSU trends in the study region while accounting for natural interannual variability across the time series (1961–2023). All GAMs were fit using a Gaussian distribution. We modeled interannual trends (all months) but also ran separate models for meteorological summer (June to August) and meteorological winter (December to February) to better understand seasonal trends during the hottest and coldest months. We determined the average rate of change (°C or PSU/decade) across the study period (1972–2023) by calculating the mean of the first derivative for each year across the GAMs smooth term.

Beyond our expectation of increasing SST averages, we also expected the frequency and duration of extreme temperatures during the summer to have increased, potentially playing an important role in kelp losses. We therefore calculated the number of days per year above both 18°C and 20°C, two critical thermal thresholds for *Nereocystis* stress and reproductive failure, respectively (Fales et al., 2023; Lüning & Freshwater, 1988; Schiltroth, 2021; Weigel et al., 2023). We used logistic regression to test whether years containing extreme heat events (both 18°C and 20°C) had become more common over the Chrome Island time series. Recognizing the many co‐occurring variables that affect kelp persistence, we used fairly conservative definitions for extreme heat events, defining a “stress event” as 2 weeks of consecutive days with SST either at or above 18°C (Lüning & Freshwater, 1988) and a “reproductive failure” event as seven or more at or above 20°C. Prolonged exposure at 20°C can result in mortality of both sporophytes and gametophytes (Lüning & Freshwater, 1988; Weigel et al., 2023) while *Nereocystis* zoospores undergo lysis within 24 h at this temperature (Schiltroth, 2021). We used Mann–Kendall tests and Sen's slope analyses to determine whether the annual number of extreme heat days had increased over time, and by how much (Amos et al., 2015). Finally, to characterize the relative decrease in water temperatures within the surveyed depths of the water column compared to those at the surface (e.g., those measured at Chrome Island), we also measured water temperature profiles monthly in June and July 2024 at four known historical kelp locations along the shelf near Cape Lazo (Appendix S1: Table S1).

While the daily time series from Chrome Island allowed for detailed insight into oceanographic trends in the study region over the last half‐century, understanding these trends within the broader context of long‐term historical climate change in the Salish Sea and beyond was also important, as natural variation from climate cycles, such as the PDO, may otherwise obscure long‐term trends. Moreover, climate reconstructions for the Pacific Northwest of North America have shown that warming trends over the past half century are unprecedented, and historical temperature averages over the past thousand years have been more similar to those in the early 20th century (Heeter et al., 2023). To contextualize the oceanographic trends seen in our study region over the past half‐century within the broader long‐term trends in the Salish Sea, we used SST records from all four active government lighthouses in the region (Entrance Island: 1936–present; Departure Bay: 1915–present; Race Rocks: 1921–present; Chrome Island: 1962–present), for which multidecadal SST trends are highly correlated with one another (Amos et al., 2015). We also used open data from the National Oceanic and Atmospheric Administration's Extended Reconstructed Sea Surface Temperature (Version 5) to plot average global SST anomalies from 1880 to 2023 as well as corresponding annual averages of the PDO index. The global SST anomalies were calculated using 1971–2000 as the baseline for depicting change (Huang et al., 2017). We used a GAM to create a composite model of SST anomalies in the Salish Sea dating back to 1915 using the same 1971–2000 baseline for ease of comparison with global anomalies. We used Pearson's correlation coefficient to determine the correlation of Salish Sea anomaly trends with both the global SST anomalies and the PDO index.

### Changes to *Nereocystis* extent over a half century

We investigated changes to the surface‐canopy forming foundation species, *N. luetkeana* (hereafter, *Nereocystis*), by comparing the extents of modern (2023) and historical (1972) surface canopies. Historical extent was delineated from aerial imagery originally collected in 1972 with the purpose of mapping nearshore seaweed communities in a series of un‐digitized reports to Fisheries and Oceans Canada, with a focus on the red algae *Mazzaella splendens*. A small subset of these data were also published in a methodology focused journal article (Austin, 1978). We discovered the un‐digitized reports and accompanying unpublished historical aerial photographs in the University of Victoria archives. The historical photographs were collected from a fixed‐wing aerial survey on August 6th and 7th, 1972. The survey covered a 2.1‐km swath along the entire ~57‐km shoreline of the 1972 study region (Appendix S1: Figure S1) during low tides (~0.6 m above chart datum, which is based on lower low water, large tide [LLWLT] in Canada). Nadir facing photographs were taken using both color and black/white (b/w) film at an approximate scale of 1:10,000 (Austin, 1978). We scanned, digitized, and georeferenced the historical photographs using Helmert transformations in QGIS software (v3.30), then manually drew polygons around the edges of *Nereocystis* surface canopy, which was easily distinguishable in both the color and b/w photographs at 1:10,000. In locations where *Nereocystis* was sparse, we used a general rule that any kelps within 10 m of one another were considered as continuous canopy extent; however, the vast majority of kelp canopy in the imagery was dense and continuous. The introduced canopy forming species *Sargassum muticum* was also present along the shallower edges of the shoreline, and differentiation of the two species was aided by the algal vegetation maps created by the original survey team, who used ground truth transects and boat based observations to delineate the dominant species or species groupings present in the historical imagery (Austin, 1978).

During the 1972 flights, additional color photographs at 1:5000 and 1:2500 scales were taken over ~10 km of the shoreline at a width of ~500 and ~250 m, respectively. The higher resolution images collected just north of Cape Lazo show small patches of *Nereocystis* that could not be seen in the 1:10000 photographs and were not delineated on the algal vegetation maps (Appendix S1: Figure S2). We chose to include these patches in our historical polygons as the characteristic *Nereocystis* bulb and blades were distinguishable from *Sargassum* canopy. A second aerial survey (1:10000 scale color photographs) was conducted in 1974, extending northward from Miracle Beach up to Campbell River and multiple smaller islands in the northern Strait of Georgia (Appendix S1: Figure S1). However, conditions during these flights were unfavorable for detection of *Nereocystis* canopy and we were unable to verify the extent or location of *Nereocystis* canopies delineated on the associated 1974 vegetation maps. Moreover, the 1974 surveys did not use the same ground truth methods so subtidal community data were not directly comparable between surveys. For these reasons, we did not include the 1974 surveys in our *Nereocystis* change analyses; however, we did resurvey a portion of the 1974 region to supplement qualitative information about modern *Nereocystis* distributions in the Strait of Georgia.

To generate a high‐resolution map of modern *Nereocystis* distribution in the northern Strait of Georgia, an aerial, fixed‐wing survey was conducted by the Hakai Institute's Aerial Coastal Observatory (ACO) on July 3, 2023 (0.0–0.5‐m tidal height). Two PhaseOne cameras (iXU‐RS1000, Rodenstock RS‐50 mm/Aerial (50 mm)) collected concurrent color (red, green, blue) and near‐infrared (NIR) images. A four‐band georeferenced orthomosaic was generated using structure‐from‐motion software (Agisoft Metashape Professional, V 2.1.1.). To classify floating canopy extent within the image area, classification was carried out using a freely available machine learning tool which automates kelp surface‐canopy detection in high‐resolution (<0.1 m) imagery (Denouden & Reshitnyk, 2025). Outputs from the tool were reviewed manually and areas where *S. muticum* canopy was present were identified and removed using a combination of visual identification in the high‐resolution imagery and ground surveys.

Given the highly variable nature of *Nereocystis* dynamics, we also reviewed a satellite‐derived time series of annual maximum surface‐canopy kelp extent (Bell et al., 2023) to better understand the timing of presence and/or absence of the largest *Nereocystis* forests off Cape Lazo and Denman Island between modern (2023) and historical (1972) aerial imagery. Time‐series kelp canopy data were generated using 30‐m resolution Landsat imagery (1984–2024), including from the Thematic Mapper sensor (TM: Landsat 4/5), the Enhanced Thematic Mapper Plus (ETM+; Landsat 7), and Operational Land Imager 1 and 2 (OLI 1/2; Landsat 8/9) following methods described in Bell et al. (2023). Given the spatial resolution of the imagery, these data are most suitable for detecting kelp forest canopy present in larger offshore beds (Cavanaugh et al., 2021). Our Landsat imagery dataset was subset from part of a larger, BC‐wide, and global kelp mapping effort (L. Reshitnyk, unpublished data). We also manually inspected 26 cloud‐free Landsat 1–3 Multispectral Scanner (MS) images (Appendix S1: Table S2) collected between June and September 1972–1982, although we were less confident in our ability to detect the canopies with these sensors because of the inherent issues with their low signal‐to‐noise ratio, as well as their 60‐m spatial resolutions. For this reason, long‐term satellite time series for kelp forests generally start in 1984 at the onset of the Thematic Mapper sensors (Bell et al., 2015; Bell et al., 2020; Cavanaugh et al., 2011).

### Changes to benthic kelp forest community composition over a half century

Changes in the community composition of benthic macroalgae were investigated by resurveying 65 large subtidal quadrats across 20 of the 22 transects that were originally surveyed perpendicular to shore to ground truth the aerial imagery in 1972. Of the two transects we did not resurvey, one could not be accessed because it was on private property, and the other's location was not sufficiently documented. The original transects were meant to capture the full range of substrate and associated macroalgae communities along the shoreline of the survey region (bedrock, cobble, sand, etc.) and used distinct and immovable geological features (e.g., large glacial radicals or bedrock features) to set the angle of the transects along the beach from the upper intertidal zone, down to a maximum of 6.2 meters below chart datum. For this study, we focused only on the subtidal benthic community (below 0‐m chart datum, LLWLT).

Historically, 2 × 5 meter large “quadrats” were surveyed in 50‐m intervals along each transect, using a premeasured rope and buoy system to mark the 50‐m distances between quadrat placements. The historical methods resulted in a range of depths being surveyed, dependent on the bathymetry of each transect, with a bias towards shallower quadrats (Appendix S1: Figure S3). Substrates within each quadrat were recorded as any combination of sand (<1.6 mm), pebbles (1.6 mm–6.35 cm), cobbles (6.35–25.4 cm), boulders (>25.4 cm), or bedrock, but did not contain percentages of each substrate type. Percent cover of each algae species present was recorded using a modified Braun‐Blanquet scale (76%–100% = 5, 51%–75% = 4, 26%–50% = 3, 6%–25% = 2, 1%–5% = 1, <1% = +), allowing total cover to sum above 100% due to overlapping canopy and understory species. While the historical surveys included *Nereocystis* cover in their quadrats when present, the original focus of the surveys was not on this species, and as such the SCUBA transects were often adjacent to, but rarely within, the documented *Nereocystis* beds.

We used markings on the historical vegetation maps and aerial photographs, as well as associated notes and metadata describing features along the transects to determine precise GPS coordinates of the historical transects and quadrats. Quadrat depth and substrate types were verified in situ to ensure a reasonable match with historical conditions. If the depth at our modern GPS point did not match the recorded historical depth, it was assumed that either the benthos had shifted (i.e., sandy bottom), or that the original surveyors had miscalculated the appropriate location of the quadrat along the transect using their rope‐buoy system. If the historical and modern depths did not match, the quadrat was shifted along the transect to match the correct depth. If the historical and modern substrates did not match at the correct depth, divers swam along the bathycline to the nearest position with matching substrate. Surveys were planned during low tides to maximize the number of quadrats surveyed by wading in shallow water with mask and snorkel, but SCUBA was used to survey any quadrats that were too deep to access in this manner. Substrate and vegetation cover were recorded following the same methods as historical surveys, and all cover values were converted to the mean value of each bin (e.g., 76%–100% ➔ 0.88).

Of the 65 subtidal quadrats, 17 contained only sand as a substrate at either historical and/or modern timepoints and were therefore either bare or contained eelgrass (*Zostera marina*). These quadrats were dropped from the analyses as either their cover type remained stable (total sand or full eelgrass cover) or, for some shallower quadrats within patches of eelgrass along exposed and dynamic sandy shoreline, the sand had shifted so that change in cover was determined to be from movement of sand within the area. There was no indication of declining eelgrass in the large, deeper beds, where eelgrass was historically dominant. In total, we used 48 paired quadrats with both historical and modern timepoints for the community change analyses.

Species listed in the historical surveys often used outdated nomenclature, with some species undergoing multiple taxonomic updates over the past half‐century. We used Algaebase (https://www.algaebase.org/) to update and match nomenclature in historical and modern observations. Before conducting analyses, we split all 52 species that were documented in either historical or modern surveys into functional groups (Appendix S1: Table S3) to understand broad‐scale shifts across similar groups of species. For native species, all large brown algae (*Laminariales* and *Desmarestiales*) were grouped colloquially as “kelps,” while we split all other native macroalgae into “red blades,” “green blades,” “thick turfs,” “thin turfs,” and “articulate corallines.” We also chose to group “introduced species” (*Sargassum muticum, Mazzaella japonica*, and *Lomentaria hakodatensis*) separately from native species as their status was of general interest to us (Britton‐Simmons, 2004; Saunders & Millar, 2014; South, 1968). The historical surveys did not document crustose algae of any type, and therefore, we did not document them in modern surveys.

We fit a series of GAMs (mgcv package in “R”) to characterize changes in the cover of all macroalgae combined, as well as the cover of different functional groups, across depths between historical and modern surveys. We used “cover” as the response variable and included “year” as a fixed effect, a smoothing term for “depth,” stratified by year, and a random effect for “quadrat” to account for the paired nature of observations. Models were fitted using either a gamma distribution with a log link, or a Gaussian distribution with a log transformed (log(percent cover +1)) response variable. These distributional families were selected based on the distribution of the raw data and visual inspection of diagnostic plots (Q–Q plots, residual vs. fitted values, and histograms of residuals). For gamma models, percent cover values of 0 were converted to 0.001 to satisfy the model assumption of positive values. Gamma models were used for cover of “total macroalgae,” “kelps,” “red blades,” and “thick turfs,” as cover data for these groups were strongly right skewed and gamma model residuals showed better homogeneity of variance than Gaussian alternatives. For the “introduced species” and “green blades” groups, Gaussian models were chosen as the log transformation was effective for normalizing residuals, since these data contained fewer extreme values. Remaining functional groups were not abundant enough to model at any timepoint and were removed from further analyses. Model fits were visualized on the untransformed scale to allow for intuitive comparison. If a quadrat had less than 1% cover for a particular group in both the modern and historical surveys, we excluded the quadrat from that groups model. To investigate changes in cover across depth and time, we extracted estimated marginal means from models of each functional group (using emmeans package in “R”) and used Walds *t*‐tests to compare predicted cover between timepoints across all depths as well as at depth‐specific (0.5‐m interval) intervals.

To further investigate restructuring of the subtidal macroalgal community over time, we calculated a Bray–Curtis dissimilarity matrix using untransformed percent cover data to emphasize changes in the abundance of dominant species. We then used permutational multivariate analysis of variance (PERMANOVA) to test whether community composition shifted between 1972 and 2023. The assumptions of homogenous dispersion for PERMANOVA were verified by performing ANOVA on the beta diversity between groups. We used a distance‐based redundancy analysis to visualize the shift in community composition over time and ran a SIMPER analysis to quantify which species were most contributing to community dissimilarity between years. These community shift analyses were performed using the vegan package in “R”.

We then used the CTI to quantitatively assess shifts in the thermal affinity of the macroalgae community between timepoints. First, we determined the average temperature experienced across each species' range, also called the species temperature index (STI), for all species with at least 5% cover within any single quadrat surveyed, removing rare species (Bowler & Böhning‐Gaese, 2017; Devictor et al., 2012). We determined each species' range along the coast using the University of British Columbia e‐flora database (https://ibis.geog.ubc.ca/biodiversity/eflora/), which includes observations from the Global Biodiversity Information Facility database (https://www.gbif.org/). Since some species surveyed are cosmopolitan and known to have distinct subpopulations, we restrained our distributions to the west coast of North America, between the Gulf of Alaska and Central America (Devictor et al., 2008). We then determined each species' STI using a mean sea surface temperature raster (2000–2019) from Bio‐oracle (https://www.bio-oracle.org/), clipped within 15 km of the western North American coastline and binned latitudinally (every 0.1°) to remove bias towards regions where the coastline extends longitudinally. Finally, we calculated the CTI for each quadrat at both timepoints, with each species STI weighted by their abundance in the quadrat. We used the quadrat level CTI values to fit a GAM with CTI as the response variable and included “year” as a fixed effect, a smoothing term for “depth,” stratified by year, and a random effect for “quadrat” to account for the paired nature of observations. For this GAM, we used a Gaussian distribution family and checked all assumptions with visual inspection of diagnostic plots. We determined the average CTI shift by calculating the estimated marginal means, integrated across the depth range of the fitted GAM.

To investigate the processes underlying shifts in the CTI of the macroalgae community (Figure 1), species were designated as either warm affinity or cold affinity following McLean et al. (2021). First, the mean thermal affinity of the historical community was calculated as the mean of all STI values from species that were documented in 1972. Then, all species (documented in either historical or modern surveys) with STI values higher than the mean thermal affinity of the historical community were designated as warm affinity, and all species with STI values lower than the mean thermal affinity of the historical community were designated as cold affinity. If the cover of a warm‐affinity species increased, that species contributed to tropicalization of the community, and if their cover decreased, they contributed to detropicalization of the community (Figure 1). Similarly, if the cover of a cold‐affinity species increased, that species contributed to borealization of the community, and if their cover decreased, they contributed to deborealization of the community (McLean et al., 2021). In CTI calculations, the STI of each species is relatively weighted by the species' abundance. Here, since historical survey efforts were biased towards shallower depths, shallow species were more abundant relative to deeper species. To account for this bias, we grouped quadrats into 1‐m‐depth bins and determined the change of cover for each species as a proportion of the total cover change. We then determined the proportional contribution of each thermal process to the total CTI change, allowing us to understand the relative contribution of each thermal process across all depths surveyed.

## RESULTS

### Environmental change

**FIGURE 2:**
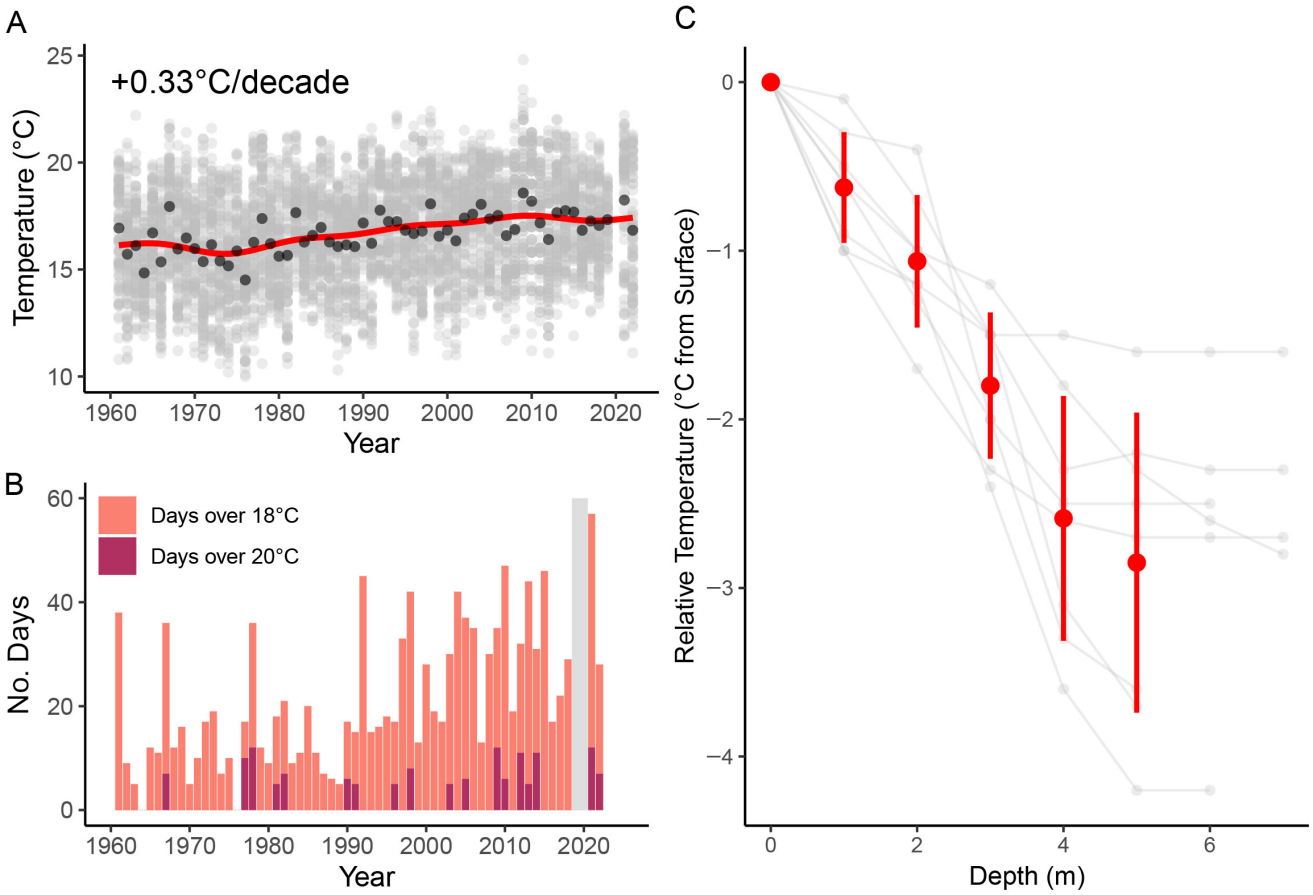
Temperature trends within the Strait of Georgia. (A) The red line shows an increasing trend of 0.33°C per decade on average between 1972 and 2023. Opaque gray points represent daily summer (June–August) temperatures at Chrome Island from 1960 to 2022, while black points show annual average temperatures. (B) Annual number of days exceeding critical temperature thresholds for *Nereocystis* at Chrome Island (June–August), with both # of days over 18°C (pink) as well as the proportion of those days that were above 20°C (purple) displayed for each year. The shaded gray box highlights that no data was collected in 2019–2020. (C) Vertical temperature profiles relative to surface temperature, recorded at four stations in 2024 located within historical *Nereocystis* forest extents (benthos approx. five meters depth below chart datum). Red markers indicate the mean temperature ± standard deviation relative to the surface temperature for each depth with the individual profiles in gray. Note that some profiles are shown extending down to seven meters depth, indicating that tidal heights during sonde casts had increased the depth between the surface and benthos.

Over the past half century, summer SST at the Chrome Island lighthouse increased by 0.33°C/decade and a total of +1.66°C (95% CI: 1.20 to 2.13°C) (Figure 2A). Summer SST warmed at nearly triple the rate of winter SST (+0.13°C/decade), while annual temperature increased by 0.25°C/decade (Appendix S1: Table S4). In comparison, global “hotspots” (i.e., the top 10% of fastest warming ocean regions globally) are considered to be oceanic regions warming faster than 0.148°C/decade (Hobday & Pecl, 2013). Conversely, annual salinity decreased by −0.20 PSU/decade annually across the study period (−1.04 PSU overall; 95% CI: −1.22 to −0.86 PSU); however, this trend was driven by decreases in winter salinity (−0.36 PSU/decade), which had decreased by nearly triple the rate of summer salinity (−0.15 PSU/decade) (Appendix S1: Table S4). Since the start of the Chrome Island time series, the frequency of “stress events” for Nereocystis (≥18°C for 14 days) increased over time (*p* = 0.009), with the length of stress events increasing by an average of 0.36 days per year (*p* < 0.001, τ = 0.34) and the odds of a stress event occurring increasing by 4.4% year‐over‐year (Figure 2B). Conversely, the frequency of mortality events (≥20°C for 7 days) remained steady over time (*p* = 0.374), although there was a weak significant trend for the increasing length of mortality events when they occurred (*p* = 0.049, τ = 0.2). Notably, at the onset of the positive shift in the PDO cycle in both 1977 and again in 1978, temperatures reached above 20°C for more than 10 days, marking the only point in the Chrome Island time series that two consecutive years contained these levels of extreme temperatures (Figure 2B). Our sonde profiles in the nearshore subtidal zone showed that by five meters depth (the approximate depth of the benthos at a tidal height of zero meters LLWLT), temperatures had decreased by an average of 2.9°C relative to surface temperatures (Figure 2C). In some profiles, temperatures decreased steadily until reaching the benthos, while in others, a stratified layer of cooler water began at around three to five meters depth, with water temperatures remaining fairly stable down to the benthos.

**FIGURE 3:**
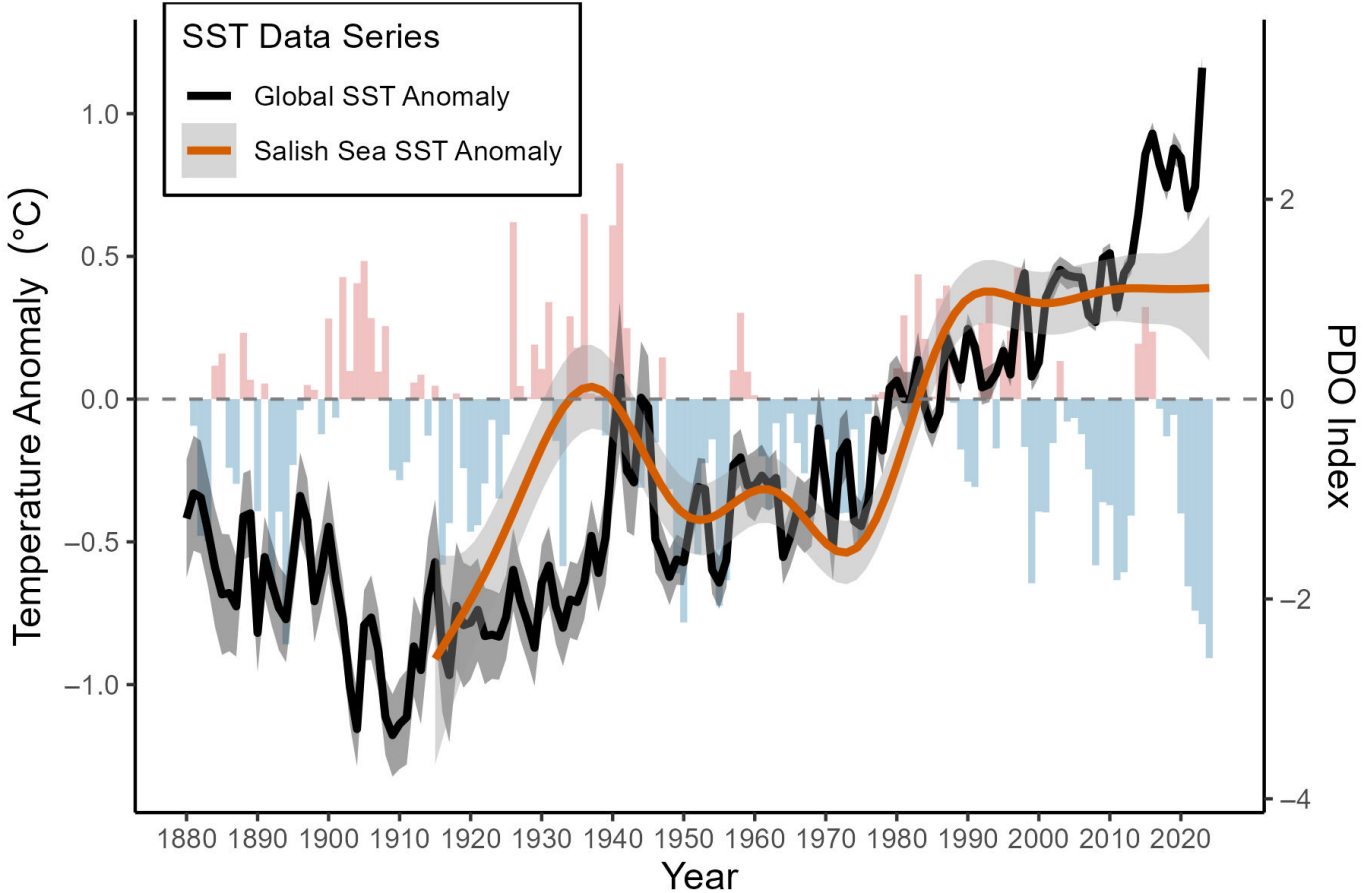
Reconstructed sea surface temperature (SST) anomalies from both global oceans and the Salish Sea, overlaid on annual pacific decadal oscillation (PDO) index values. While Salish Sea SST trends are generally modulated by positive and negative phases of the PDO, external forcing from climate change has continuously warmed global oceans over the past century, resulting in weakened modulation by the negative PDO phase and an overall increase to SST in the Salish Sea.

More broadly, annual SST trends across the Salish Sea (Figure 3) showed strong correlation with the PDO index up until around 1990 (*p* < 0.001, *r* = 0.688). The two main periods of increasing SST anomalies in the Salish Sea occurred roughly between 1915 and 1935, and again in 1975–1990, both coinciding with shifts from negative to positive PDO phases (Figure 3). Between these two periods of warming, a negative PDO phase was dominant, and SST remained largely negative relative to the 1971–2000 baseline. Yet since 1990, the PDO index has largely remained in a negative phase, reaching record lows in 2024, and Salish Sea SST has remained at record highs (Figure 3). The decoupling of recent SST trends from the PDO index add to the growing evidence of a weakened linkages between regional temperatures in the North Pacific and broad‐scale climate indices like PDO in the face of climate change (Bell et al., 2020; Kuroda et al., 2020). Moreover, the strong correlation of Salish Sea SST with continually increasing global SST since 1915 (*p* < 0.001, *r* = 0.722) suggests that the well‐established external anthropogenic forcing in global oceans (Athanase et al., 2024; Hansen et al., 2006; Marcos et al., 2025) is driving the breakdown of internal modulation from regional processes like PDO. Given the strong, long‐term correlation between the Salish Sea and global ocean temperatures, it is reasonable to infer that Salish Sea temperatures also remained relatively low from 1915 back into the late 19th century, similar to that of global oceans (Figure 3). By the time of the 1977–78 extreme heat events, annual SST in the Salish Sea had increased by 0.52°C (95% CI: 0.12–0.92°C) versus 1915.

### Changes to *Nereocystis* extent over a half century

**FIGURE 4:**
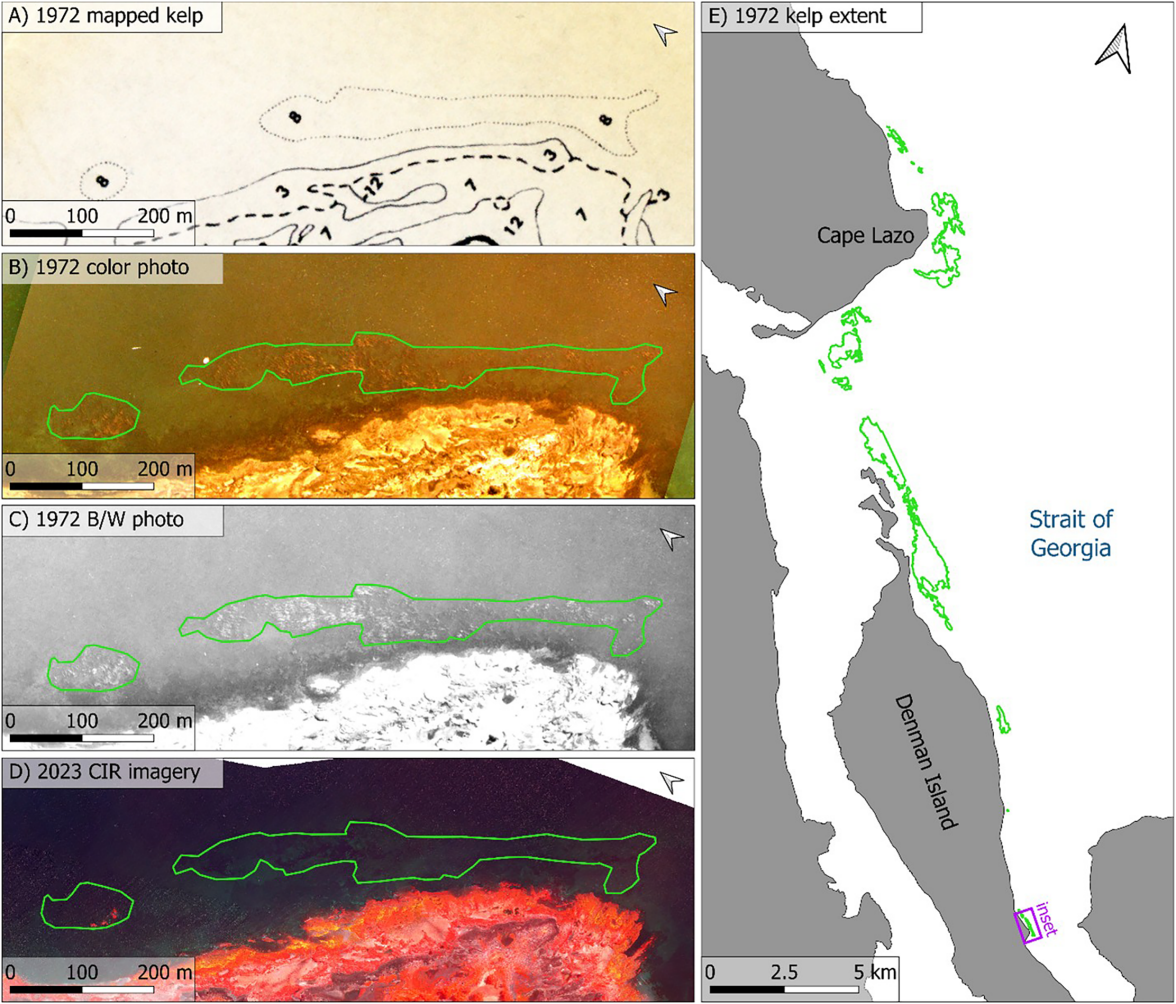
Historical bull kelp (*Nereocystis luetkeana*) surface‐canopy extent, as seen in (A) historical vegetation maps created from 1972 aerial and ground truth surveys. The historical aerial photographs were (B) color and (C) black and white photographs, both seen here with overlaid manual delineations (green polygons) of 1972 *Nereocystis* surface‐canopy extent. The same location, (D) surveyed in 2023 with color‐infrared imagery, showed that *Nereocystis* is no longer present in the delineation of historical surface‐canopy extent. Note the small patch of vegetation on the intertidal rock within the green polygon to the left of panel D, demonstrating how easily the NIR reflectance of vegetation can be contrasted with the surrounding water when present. (E) The entire *Nereocystis* extent within the survey region can be seen along Cape Lazo and Denman Island. Note the location of inset panels A–D in the purple rectangle.

Between 1972 and 2023, *Nereocystis* was completely lost from the entire 57‐km shoreline of the study region in the Strait of Georgia. In total, there were 552 hectares of *Nereocystis* surface canopy documented in the historical aerial imagery, while modern imagery showed that by 2023, no *Nereocystis* remained. Of the historical *Nereocystis* canopy, 95.4% was located between 2 and 10 m (chart datum) with the vast majority of historical canopies located along the large shelves around Cape Lazo and the northeast side of Denman Island (Figure 4). Historical *Nereocystis* was never documented deeper than the 10‐m bathycline, and only 4.6% was shallower than 2 m. The intertidal and nearshore focus of the historical aerial photographs meant that some deeper portions of the extensive shelves along the north side of Denman Island were not captured in the photographs, and in some cases dense, clearly visible *Nereocystis* canopy extended beyond the edges of the photograph on the deeper side, indicating that the total historical *Nereocystis* canopy baseline was larger than the 552 ha documented here.

*Nereocystis* canopy detected to the north of the study region in the extended 2023 imagery (Appendix S1: Figure S1) suggests that further north within the Strait of Georgia *Nereocystis* remained relatively stable between timepoints. However, even when we included these stable *Nereocystis* beds in our *Nereocystis* change analysis, roughly doubling the total shoreline length surveyed, the total decrease in extent between timepoints was still approximately 97%, demonstrating the extreme scale of historical losses on the shelves around Cape Lazo and Denman Island.

Our complementary analyses using the 30‐m Landsat satellite‐derived time series detected zero kelp forest canopies offshore from Cape Lazo or Denman Island between 1984 and 2024 (Appendix S1: Figure S4). Furthermore, we were unable to detect *Nereocystis* in any Landsat MSS images (1972–1982) (Appendix S1: Table S2), although it is suspected that this lack of detection is likely due to the poor spatial and spectral resolution in the imagery, as three of the images were captured in the summer of 1972 when large dense areas of *Nereocystis* surface canopy are otherwise clearly visible in the historical aerial imagery. Together, these results suggest that the largest *Nereocystis* forests off Cape Lazo and Denman Island were lost prior to 1984, likely during extreme marine heatwaves during the late 1970s and early 1980s, when summer temperatures (Figure 2b) surpassed the critical thresholds for *Nereocystis* growth and survival (Fales et al., 2023; Weigel et al., 2023).

### Changes to benthic kelp forest community composition over a half century

**FIGURE 5:**
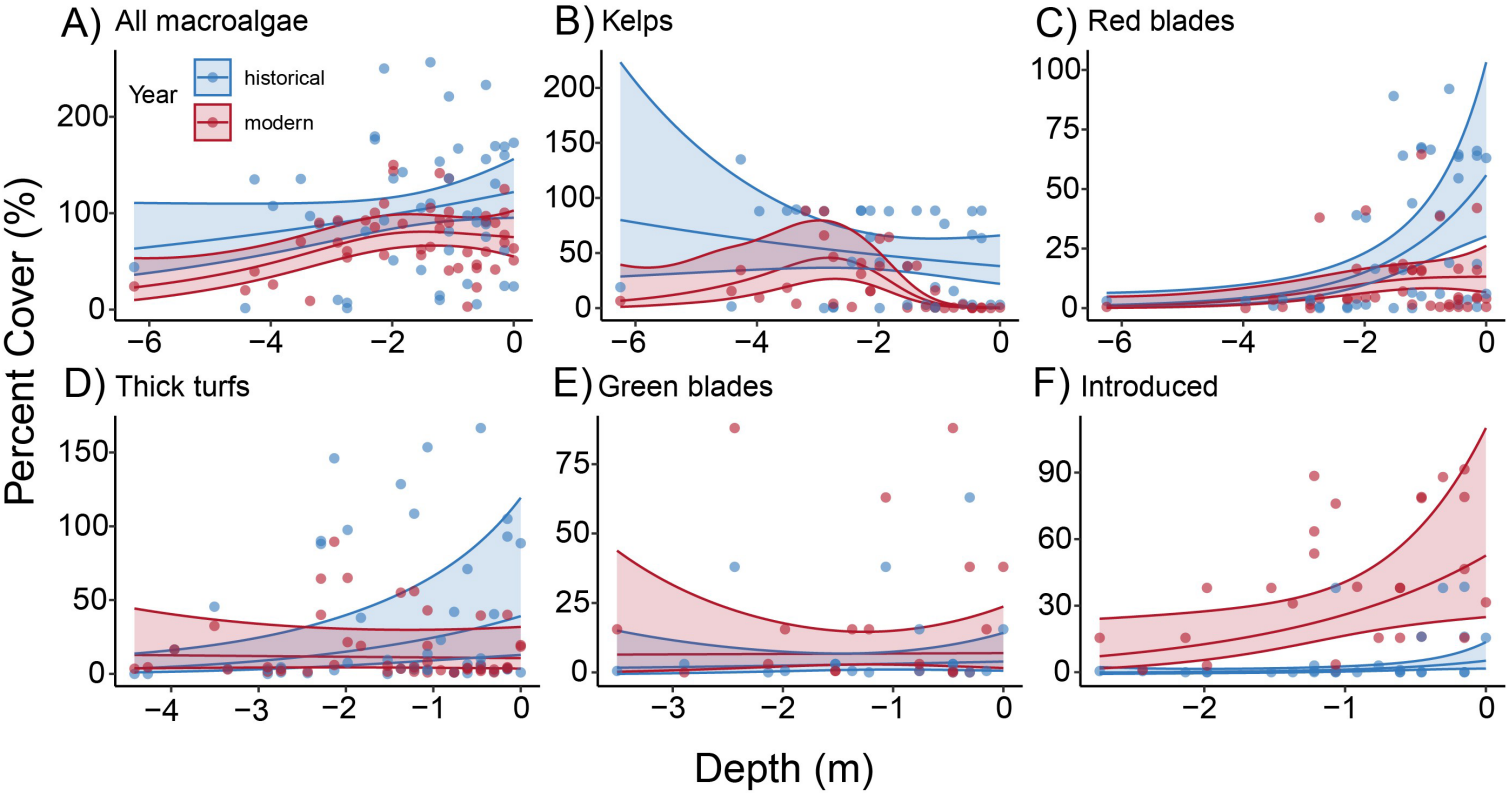
Change in total macroalgae cover and functional group cover. (A) Cover change of all combined species are grouped and modeled using GAM models, while (B–F) different functional groups are individually modeled. Note that *x* and *y*‐axes differ between plots, as trends are plotted over the depth ranges of respective functional groups, and that some quadrats, especially historically, had summed cover greater than 100%.

Our models indicate that historically, different functional groups were uniquely distributed across depths, and that change in total cover of each group between years was depth dependent (Figure 5). When all functional groups were combined, estimated marginal means showed that between 1972 and 2023, average macroalgae cover decreased by 21% across all depths, yet these decreases were significant (*p* < 0.05) only above 1.5 m depth and below 3.5 m depth. At the functional group level, the largest changes were in the shallow subtidal zone, where kelps and red blades decreased significantly above 1.5 m depth (*p* < 0.05), and thick turfs decreased significantly above 2.0 m depth (*p* < 0.05). In contrast, introduced macroalgal species increased significantly above 3.5 m depth (*p* < 0.05). Below 4.5 m depth, decreased macroalgal cover was almost exclusively due to kelp losses, although around 3 m depth, kelp species remained abundant in some quadrats, indicating spatial variability in the stability or decline of kelps between transects. Notably, historical transects rarely overlapped with *Nereocystis* beds, and therefore, virtually none of the change (or lack thereof) seen in the GAM can be directly attributed to the extirpation of Nereocystis. Green blades had no significant changes at any depths. Overall, while this functional group approach provided some insights regarding community changes, species level analyses were more revealing. For example, in some cases “kelp stability” was driven by the replacement of *Saccharina latissima* with *Neoagarum fimbriatum*, which was not detected in any historical surveys but became dominant in some quadrats, indicating that the normally deeper *N. fimbriatum* may be moving shallower in the subtidal zone.

**FIGURE 6:**
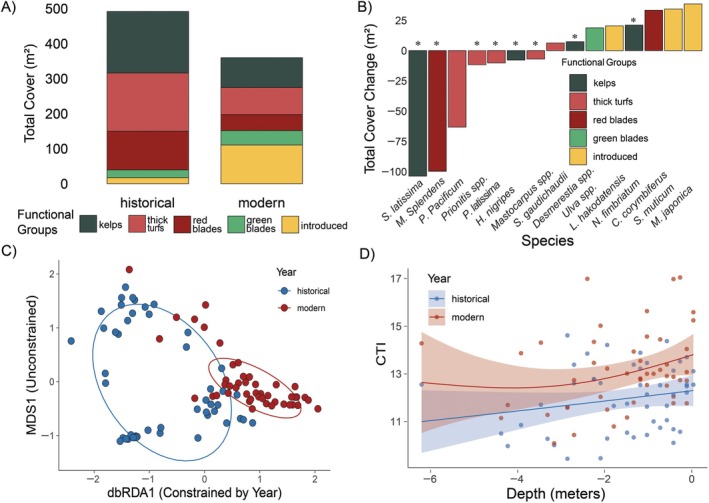
Changes to subtidal macroalgae community composition between 1972 and 2023. (A) Total cover of each functional group in historical and modern surveys, showing the relative cover change of each group between the two survey timepoints. (B) Total cover change between the survey timepoints for the 15 macroalgal species with the largest absolute change, color‐coded by functional group. An asterisk (*) above the bar denotes that it is a cold‐affinity species. (C) Distance‐based redundancy analysis (dbRDA) plot showing substantial shifts in composition across the constrained axis (Year) between historical (blue) and modern (red) communities. (D) Quadrat level community temperature index (CTI) values paired for historical (1972—blue) and modern (2023—red) surveys, with generalized additive models for each timepoint overlayed showing an increase in CTI across all depths.

Overall, across all quadrats surveyed, decreased macroalgal cover was not only due to declines in kelp species, but also to losses of other dominant functional groups, including red bladed algae and thick turfs, offset by increases in introduced species (Figure 6A). The decreased total cover of macroalgae at certain depths was driven by decreased cover of the three most historically dominant species (Figure 6B), the kelp, *S. latissima* (−78%), the red bladed algae, *M. splendens* (−98.5%), and the thick turf, *Plocamium pacificum* (−62.1%). The total cover of the introduced brown algae, *Sargassum muticum*, increased the second most of all species (Figure 6B) and became the most abundant species detected in modern surveys with a relative increase of 304%. Total cover of the introduced red algae, *Mazzaella japonica*, increased the largest amount (Figure 6B), becoming the third most abundant species after not appearing in any historical surveys. Although the cover of some warm‐affinity native species, like the red bladed algae *Chondracanthus corymbiferus*, also increased, overall, the species that increased were greatly outweighed by the loss of historically dominant species.

The PERMANOVA showed that community composition was substantially restructured over the past half‐century (Pr(>F) 0.001), which is apparent by the dissimilarity seen between the modern and historical quadrats on the constrained axis of the distance‐based redundancy analysis plot (Figure 6C). Declines in the three most historically dominant benthic species, *S. latissima*, *M. splendens*, and *P. pacificum*, contributed to 42.7% of community dissimilarity between timepoints (Appendix S1: Table S5), while the species that increased only accounted for 20.1% of the dissimilarity. Between 1972 and 2023, the CTI increased by 1.4°C (95% CI: 0.43–2.37°C) (Figure 6D) when averaged across all depths (*p* < 0.001), which means that the macroalgal CTI has been tracking increases in summer water temperatures (+1.66°C; 95% CI: 1.20–2.13°C) more closely than increases in winter temperatures (+0.65°C; 95% CI: 0.46–0.84°C).

**FIGURE 7:**
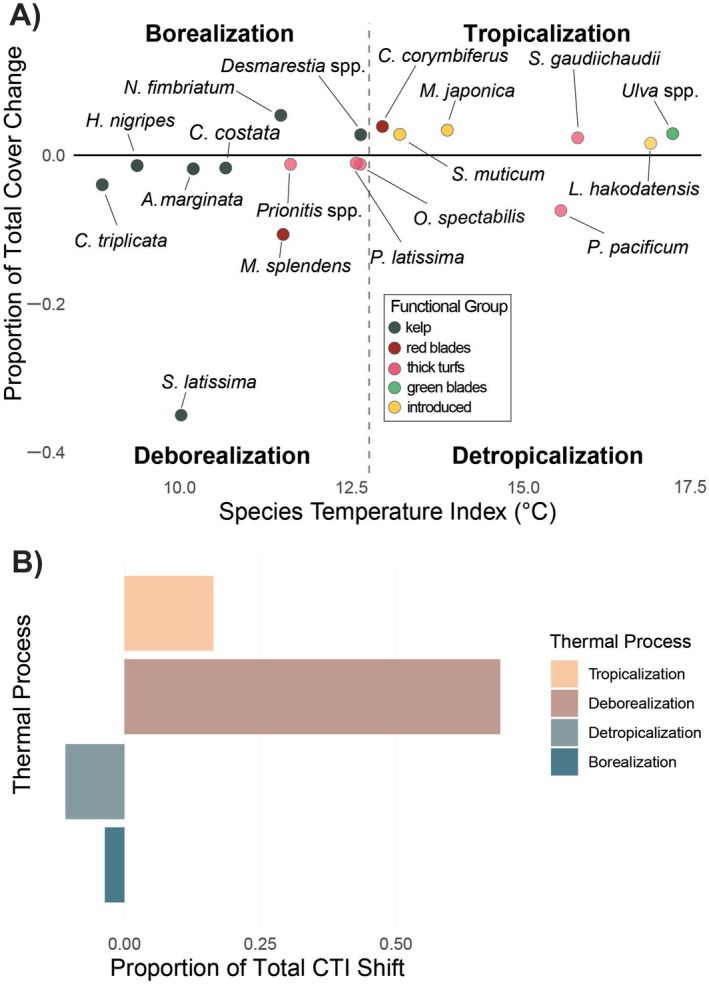
Species' contributions to thermal processes and associated Community Temperature Index (CTI) shift. (A) Species' contributions to a thermal process are determined by their change in cover and their thermal affinity (i.e., species temperature index). The four quadrants shown on the plot are identical to those in Figure 1, although here, the dashed gray line on the *x* axis is the mean thermal affinity of all species in the historical macroalgae community. Species to the left of the dashed gray line are “cold affinity” and species to the right of the dashed gray line are “warm affinity.” Each species is colored according to functional group, and only species that contributed to more than 5% of total cover change after depth‐averaging are shown. (B) The proportion that each thermal process has contributed to the overall CTI shift since between 1972 and 2023, with deborealization as the dominant process.

Examining CTI across all depths equally, deborealization was by far the strongest contributor to the CTI increase overall (Figure 7 A‐B). Even in the shallowest waters (<3 m depth), where introduced and native warm‐affinity species both increased the most, deborealization associated with the loss of the cold‐affinity *S. latissima* and *M. splendens* strongly outweighed tropicalization. With the bias towards shallow quadrats accounted for, the contributions of historically common deeper kelps (>3 meters depth) towards community deborealization were weighted more evenly (Figure 7A) to shallower species that had a similar change in abundance, but were more commonly detected due to their shallower distributions.

## DISCUSSION

Our analysis of half‐century‐old aerial imagery increased the baseline for extent of the region's foundation species *N. luetkeana* by more than an order of magnitude and complementary satellite data revealed that these kelp forests were lost decades before recent marine heatwaves. Moreover, our SCUBA surveys showed that restructuring of this hotspot's subtidal macroalgal communities occurred mainly through deborealization, rather than the more commonly recognized process of tropicalization, suggesting that deborealization may be the dominant process restructuring macroalgal communities in rapidly warming temperate and polar waters that have low connectivity to marine regions with warmer climates. Here, deborealization was largely due to loss of dominant understory kelps, but losses of other cold‐affinity macroalgae species contributed to deborealization as well, especially within the shallowest subtidal zone (<3 m). Together, these findings emphasize the importance of historical data in setting accurate baselines for the management of coastal ecosystems and informing decision‐making related to conservation and restoration in coastal marine ecosystems (Eger, Aguirre, et al., 2023; Hollarsmith et al., 2022). Integrating a long‐term understanding of climate change and other anthropogenic impacts can avoid “shifting baseline syndrome” and improve decision‐making related to management of coastal resources.

### Changes to *Nereocystis* extent over a half century

Our use of historical aerial imagery from 1972 greatly increased the historical baseline extent for *Nereocystis* in the northern Salish Sea over previous estimates (Mora‐Soto et al., 2024a), while also providing further evidence for extirpation of foundation species within warming microclimates along temperate coastlines (Starko et al., 2022). The magnitude of the difference between our half‐century historical baseline and more recent high‐resolution satellite‐derived baselines (Mora‐Soto et al., 2024a) demonstrates the importance of integrating multiple historical data sources to understand long‐term climate impacts and associated habitat change. These differences are particularly relevant to decision‐making by marine managers, as remote sensing methodologies are being incorporated into regional, national, and even global management frameworks, including those focused on status and trends (Frieder et al., 2025), restoration needs (Giraldo‐Ospina et al., 2025), and “blue carbon” accounting (McHenry et al., 2025). Mora‐Soto et al. (2024a) also used historical nautical charts to show that kelp forests off Cape Lazo were present at some point between 1853 and 1956 (Mora‐Soto et al., 2024a), although the markings on these charts can only be used to determine the presence of kelp historically, not the extent (Costa et al., 2020). Despite the large increase of the baseline for *Nereocystis* extent that was derived from the 1972 imagery used in our study, it is likely we still underestimated the total historical *Nereocystis* extent in the region, given the additional *Nereocystis* canopy detected in the limited areas covered by larger scale 1972 photographs (1:2500 and 1:5000 scale), as well as the unmapped *Nereocystis* canopy that extended into deeper water beyond the edges of the 1:10,000 scale historical photographs.

In our analyses, the combination of Landsat satellite imagery and the SST records from the Chrome Island lighthouse suggests that *Nereocystis* populations collapsed during the shift into a positive phase of the PDO (Peterson & Schwing, 2003), with extreme summer heat occurring during 1977–1978 as well as the subsequent El Niño of 1981–1982. During these years, summer temperatures in the Strait of Georgia remained above lethal thermal thresholds for *Nereocystis* (20°C) (Fales et al., 2023; Weigel et al., 2023) for more than a week at a time. During the same time period, surface‐canopy forming kelp populations across the northeast Pacific experienced large declines and collapses from California to Haida Gwaii, BC (Berry et al., 2021; Dayton et al., 1998; Gendall et al., 2025; Tegner et al., 1996). In some parts of the northeast Pacific, kelp forests showed resilience and have since rebounded from these historical collapses (Leichter et al., 2023), while other regions experienced continuous declines and even extirpations (Berry et al., 2021; Gendall et al., 2025). We suspect that during the summers of 1977–1978, temperatures in the Strait of Georgia were lethal not only to the adult sporophytes, but also to the gametophyte “seed bank” that would have otherwise persisted on the sea floor. Loss of benthic gametophytes would have inhibited the annual re‐establishment of the *Nereocystis* in subsequent years, especially as there were multiple years in the late 1980s where cooler water temperatures would have otherwise likely allowed *Nereocystis* to flourish if the gametophyte seed bank had persisted. Together, these lines of evidence suggest that the majority of *Nereocystis* beds, specifically the largest beds off Cape Lazo and northern Denman Island, had collapsed by 1984 by the time higher resolution satellite imagery became available (Appendix S1: Figure S4). *Nereocystis* persistence to the north of our study region is likely due to influence from the cold, well‐mixed waters of the Johnstone Strait that enter the northern reaches of the Strait of Georgia.

While the extreme temperatures associated with the shift to the positive PDO phase in 1977–1978 have not been directly linked to climate change, it is well established that the warming of global oceans is amplifying marine heatwaves (Athanase et al., 2024; Marcos et al., 2025). By 1977, global SST had already been steadily climbing for roughly 60 years (Figure 3), and since then there has been an increased frequency of warm water events along the western coast of North America (McGowan et al., 1998), as well as drastic and continuous increases in land–ocean temperature anomalies globally (Hansen et al., 2006). Moreover, there has been evidence of weakened linkages between the PDO cycle with regional physical and ecological processes in recent decades (Amos et al., 2015; Bell et al., 2020; Kuroda et al., 2020). This weakening trend is apparent in the Salish Sea, where extreme summer heat has remained persistent, even during strong negative phases of the PDO cycle (Figure 3). Therefore, regardless of the role of climate change in any particular heatwave, increased summer temperatures in the Strait of Georgia (Figure 2A), which frequently surpass thermal thresholds for *Nereocystis* reproduction (Figure 2B), have likely created a persistent barrier to the reestablishment of *Nereocystis* after its initial decline.

It is also possible that smaller patches of *Nereocystis* (i.e., tens of meters, rather than hundreds of hectares) persisted in the region within small refugia, or even reestablished in some locations only to disappear again before our 2023 survey. For example, just outside our study region on the southern tip of Denman Island, a small *Nereocystis* patch was present just before, and was lost during, the “blob” marine heatwave in 2015 (Starko et al., 2024). During early summer surveys in 2023, we spotted small rafts of seemingly healthy *Nereocystis* (between 1 and 5 individuals) with reproductive sori patches drifting as far south as Denman Island, indicating some connectivity and theoretical potential for reestablishment of kelp from persistent northern populations in cooler years. Thus, while the majority of *Nereocystis* appears to have been lost nearly half a century ago, there is evidence that more recent marine heatwaves were likely the “final nail in the coffin” for any small populations of *Nereocystis* that may have remained within isolated refugia in the region as recently as a decade ago (Starko et al., 2024).

Although our modern aerial surveys showed that *Nereocystis* was lost from the region, we also detected 33.8 ha of *Sargassum muticum* surface canopy, which was almost entirely located above the 2‐m bathycline. The *S. muticum* in our modern surveys and on the historical maps only overlapped with historical *Nereocystis* in the few areas where the shoreline had a steep slope, indicating that *Nereocystis* losses were generally not due to being outcompeted by increasing *S. muticum*. To the north of our study region, near Campbell River, B.C., many of the shorelines have steeper slopes and, as a result, the remaining *Nereocystis* beds here often merge into *S. muticum* beds in shallower water. These observations are particularly important for remote sensing practitioners as well as managers using remote sensing products for habitat characterization and decision‐making, as even with the highest spatial resolution satellite imagery, differentiation between the areas of *S. muticum* and *Nereocystis* is likely not possible, and generally unaccounted for in satellite remote sensing methodologies for kelp canopy detection.

For example, we suspect that most, if not all, of the previously reported increases of *Nereocystis* along the shoreline of our study region during and after the “blob” heatwave (Mora‐Soto et al., 2024a) were in fact *S. muticum* canopies, as all of these reported increases of *Nereocystis* occurred between 0 and 2 m depth within the 2014–2022 time period (Pers. Comm. Mora‐Soto, 2025). In 2023, we saw dense *S. muticum* beds, some extending over 100 m seaward from the 0‐m bathycline, meaning there is high potential for misclassification from remote sensing data if masking only above the 0‐m bathycline, or using simple distance‐based buffers (e.g., a 30‐m buffer for Landsat). Ground truthing of satellite data and local knowledge of surveyed regions are crucially important in these contexts. To avoid false detections it may be preferable to mask out any vegetation shallower than 2 m depth, especially at lower tidal heights or if using red‐edge bands to increase canopy detection (Timmer et al., 2022, 2024). In regions where loss of canopy forming kelps is suspected, understanding the potential for these types of false detections is especially important to remote sensing practitioners as well as managers using remote sensing data for decision‐making.

The large historical *Nereocystis* beds documented herein would likely have been important nursery and foraging habitat for culturally and commercially important species, such as the numerous now‐threatened or endangered Pacific salmon (*Oncorhynchus* spp.) stocks migrating in and out of the Salish Sea (COSEWIC, 2016; COSEWIC, 2017), as well as the Pacific herring (*Clupea pallasii*) that spawn and rear in the Salish Sea (Shaffer et al., 2020; Shaffer et al., 2023). The loss of these large kelp forests may present an opportunity to enhance nearshore ecosystems through kelp restoration, with detailed historical baselines providing guidance to marine managers on which areas might be best suited to focus restoration efforts. Even if the region's warm ocean temperatures in the late summer prevent self‐sustaining *Nereocystis* populations, the fast growing and annual life history of *Nereocystis* may mean that the habitat provisioning and other ecosystem services (e.g., nutrient cycling) provided by this species could make annual restoration efforts worthwhile.

### Changes to benthic kelp forest community composition over a half century

The results of our community analyses support our hypothesis that this kelp forest community was primarily restructured by deborealization over the past half century. Between the two timepoints (1972 and 2023), the CTI of the macroalgal community increased by 1.4°C, tracking increases in summer SST (+1.66°C) more closely than winter SST (+0.65°C) and higher than annual (+1.27°C), indicating that warmer summer temperatures are likely a key driver of these community shifts. We hypothesized that the community here would be restructured by deborealization, rather than tropicalization, for two key reasons. First, we expected that extreme temperatures during summer months had surpassed the thermal optimums and thresholds of various cold‐affinity species, especially kelps and including the foundation species *N. luetkeana*, which would result in either reduced abundance or extirpation of those species (Starko et al., 2022; Wernberg et al., 2024). Since kelps are relatively large and productive compared to most algae, we expected the reduced abundance of kelp to outpace any increase of less productive warm‐affinity native species, reducing the overall vegetation cover in the region. Second, microclimates are generally disjunct from oceanographic regions that reach similar temperatures and might contain warm‐adapted populations of macroalgae, or even kelps, that would thrive under similar temperatures. We posit that the generally low dispersal abilities of macroalgae, combined with the large distances between these southern populations and our study region, explain the lack of range expansion (i.e., tropicalization by “species on the move”) that occur in transition zones closer to subtropical populations (de Azevedo et al., 2023; Vergés et al., 2014). Moreover, these dominant processes may be common within microclimates along temperate coastlines that are not subject to poleward flowing currents, for example, along the western coast of South America and portions of the northeast and northwest Atlantic coastlines (Vergés et al., 2014), making the understanding of how and why deborealization is occurring relevant to marine managers working on such coastlines in general.

While range expansion of warm‐adapted “species on the move” is one way that tropicalization may occur (McLean et al., 2021; Pinsky et al., 2013; Vergés et al., 2019), there can also be tropicalization through the anthropogenic introduction of warm‐adapted species from other parts of the world (Bianchi & Morri, 2003; Vergés et al., 2014), or simply through the increased abundance of native species with thermal affinities above the historical mean of the community (e.g., those historically present but at the poleward edges of their range) (Vergés et al., 2014). In our study, these two latter forms of tropicalization were responsible for all of the documented tropicalization. When connectivity is higher with warm‐adapted populations (either through population proximity or higher dispersal ability), tropicalization through range expansion is likely to contribute more to the overall shifts in CTI, making deborealization less dominant (Chust et al., 2024). Since each of these three forms of tropicalization has different ecological implications on the broader nearshore ecosystem (e.g., nearshore productivity, habitat complexity, species interactions), it is crucial that marine managers and those making policy decisions around conservation and restoration have a functional understanding of the differences therein. As such, we suggest that future work using CTI frameworks identify and differentiate tropicalization resulting from increased abundance of native species, human‐introduced species, or poleward range expansions.

With only two timepoints in our community comparison, we can only speculate as to the timing of abundance shifts for the other macroalgal species and their associated thermal processes. However, similar to the large differences between our long‐term *Nereocystis* baselines and more recent *Nereocystis* baselines, it should be considered that trends in community shifts and their associated thermal processes will also likely vary across different timescales. For example, recent work on the coast of Portugal has shown that between 2012 and 2018, rapid tropicalization occurred in macroalgal communities (de Azevedo et al., 2023), yet four decades of monitoring on the coast of France showed that more recent periods of tropicalization were preceded by historical periods of deborealization (Arriaga et al., 2024). Moreover, shifts in community composition may be temporary, and if extirpation does not occur, communities may return to historical states when temperatures subside, potentially masking impacts on, or interactions with, the broader marine fauna community that occurred during years of extreme temperature (Arriaga et al., 2024; Wernberg et al., 2024).

The composition of macroalgal communities can fluctuate annually, and even seasonally, and it is important to recognize that both the historical and modern surveys in our study are only snapshots in time. Without more detailed time‐series data available to understand the year‐to‐year variability in community shifts, we must turn to other indirect evidence—such as comparing magnitudes of change for different species and understanding species' life histories—to interpret the ecological relevance of the shifts that we observed. For example, species may be fully extirpated from an area, as seen for *Nereocystis* and *C. triplicata*, or may remain a persistent species in the community at a reduced depth range or abundance, like *S. latissima*. The ability for an extirpated population to reestablish depends not only on the reversal of the conditions that led to extirpation, but also on connectivity with potential source populations of the extirpated species (Maunder, 1992). During multiple SCUBA and underwater drone surveys by the lead author in the nearest healthy *Nereocystis* forest at Oyster River, about 25 km north of Cape Lazo, *C. triplicata* was abundant. Yet the lack of reestablishment for either *C. triplicata* or *Nereocystis* along the continuous shoreline southward indicates that persistent high temperatures are likely inhibiting their reestablishment.

We documented the most extreme community changes in the shallowest water (<3 m), where both historical cover and modern temperatures were the highest. Some species with a narrow depth range that were only dominant in shallow water, like the red bladed algae *M. splendens* (−98.5%), functionally collapsed across the region. The most dominant subtidal species, *S. latissima*, was historically abundant across the entire depth range surveyed, but its depth range had contracted to only the deeper water column during modern surveys. Within our study region, the subpopulation of *S. latissima* displays an annual phenology (BT personal observation), meaning that no sporophytes remain during winter months and sporogenesis must occur during summer months when hot temperatures in the shallow subtidal zone are likely to cause failed recruitment. However, the high connectivity across the short distance between shallow to deep habitat means it may only take a few colder years for *S. latissima* to become abundant in the shallow subtidal zone once again. These two examples show that depth ranges of different species may play a key role in the persistence of species within warming microclimates.

Despite being a useful characterization of community shifts under climate change, the CTI framework and its respective processes do have limitations. While increasing temperatures are well established as a key driver of composition shifts within macroalgal communities, other factors including light availability, salinity, nutrients, and interactions with other species can also influence the abundance of macroalgal species (Fragkopoulou et al., 2022). For example, in a dense kelp forest, the loss of a canopy forming species like *Nereocystis* may initially increase cover of understory species due to increased light availability. Moreover, despite the CTI framework being useful for capturing broad‐scale community trends related to temperature changes, data limitations related to fine‐scale environmental parameters or genetic variation within subpopulations may pose challenges. Indeed, despite the overall increase of CTI detected in this study, not all changes in species cover were accounted for by the processes of deborealization and tropicalization, indicating that either species have been miscategorized as either warm or cold‐affinity species, or that additional influences in the environment may have driven some of the changes in community composition. For example, rather than increasing, the “warm‐affinity” species *P. pacificum* had the third largest decrease in cover in our study, contributing to the process of detropicalization and lowering the CTI. This apparent contradiction in a warming ocean may be due to the localized heat adaptation displayed by *P. pacificum* populations from northern and southern latitudes along *P. pacificum*'s large range (Biebl, 1970). There is a growing body of research showing that macroalgae populations within a range can be locally adapted, with varying thermal limits within a species' range (King et al., 2018, 2019; Solas et al., 2023). Within the simplistic CTI framework, and without range information for these potential subpopulations, we cannot account for adaptation within local populations. However, supplementing CTI analyses with other lines of evidence may be key to a disentangling the factors restructuring marine communities. For example, thermal threshold experiments or genetic information, as well as life histories and knowledge about species interactions could be important lines of further inquiry.

Additional examples of contradictions within the CTI framework are the increases of the “cold‐affinity” kelp *Neoagarum fimbriatum* and the ligulate *Desmarestia* species, both of which were examples of borealization. *N. fimbriatum* is a perennial species that usually occurs in deeper water with relatively low productivity compared to other kelps (Bell & Kroeker, 2022), and its increased abundance in shallow waters may be a symptom of coastal darkening allowing a species with lower light optima to outcompete other shallow species that require more light (Blain et al., 2021; Frigstad et al., 2023). While there is some evidence of coastal darkening in the northeast Pacific in recent decades (Davies & Tim Smyth, 2025), focused study within the Salish Sea has been highly limited (Del Bel Belluz et al., 2021). However, if the shoaling of deeper species is truly an indication of coastal darkening in this region, we expect that there may also be negative impacts on other species, possibly co‐occurring with other stressors like temperature (Blain et al., 2021), including on *N. luetkeana*. In contrast, the ligulate *Desmarestia* species are known to be relatively fast growing and “weedy” annuals and may be simply taking advantage of the space opened by reduced macroalgae cover of other species (Edwards, 1998). These examples highlight that while CTI may be a valuable tool for general understanding climate related shifts within communities, there are multiple complex factors at play that can result in abundance and distribution shifts.

The broader consequences of kelp forest community deborealization are likely to extend into fisheries and marine ecosystem management. Poleward shifts resulting in tropicalization of temperate fish and invertebrate communities have been fairly well documented globally (Chust et al., 2024; Perry et al., 2005), yet many marine organisms are highly dependent on kelp forest ecosystems for habitat and food. If deborealized kelp forest communities are becoming less productive and are unable to support marine species that are shifting poleward, it will be crucial for fishery managers to account for reduced carrying capacities, especially in estuaries and inland seas that otherwise provide valuable nursery habitat for various marine species (Pérez‐Matus et al., 2025) but are warming rapidly (Prum et al., 2024). Moreover, if habitat provision and productivity from cold‐affinity native species are being replaced by that of introduced warm‐affinity seaweed species, there are important considerations to be made about whether facilitating introductions as a form of habitat “restoration” is viable in regions of habitat loss. While we strongly recommend focused study into the potential risks of introducing a habitat‐forming species, like *Sargassum muticum*, into a new region, such introductions could be a viable alternative to ongoing efforts using modification or selection of certain genetic traits in threatened extant populations, which come with risks of their own. We do not purport to have answers to these questions, but emphasize that discussion and understanding of these topics will be increasingly important for scientists, managers, and policymakers alike as the climate continues to warm.

## CONCLUSION

To understand the full extent of long‐term climate change impacts, historical baselines are crucial. Here, we showed that in a temperate coastal hotspot, kelp forests formed by the foundation species *N. luetkeana* collapsed decades earlier than more recent marine heatwaves, underscoring that extreme heat events have been impacting coastal ecosystems for at least the past half‐century. Further, while CTI had increased within the macroalgal community, consistent with expectation for a warming coastal ocean, the increases were primarily due to deborealization, rather than tropicalization of the community, adding to the growing body of evidence that deborealization is occurring across multiple taxa within rapidly warming semi‐enclosed basins (Chust et al., 2024). The dominance of deborealization over tropicalization within this temperate coastal hotspot was likely due to the combination of extreme temperatures, which decreased the abundance of various cold‐affinity species, as well as a lack of range expansion from disjunct warm‐affinity populations to the south. Temperate microclimates are often disjunct from regions of similar climatology where warm‐affinity populations exist, limiting potential for range extensions into the numerous semi‐enclosed basins such as fjords and inland seas (Chust et al., 2024; Starko et al., 2022, 2024) that are common beyond forty degrees latitude in both the northern and southern hemispheres (Bianchi et al., 2020). Notably, the limited tropicalization that was observed was largely due to human‐facilitated introductions. Together, these findings are relevant for informing the decision‐making of marine managers on topics like fisheries, blue carbon, restoration, and conservation as climate change continues. Deborealization within warming temperate microclimates may shift communities towards smaller and less productive species, resulting in reduced habitat complexity and reduced nutrient cycling (Duggins et al., 1989), with potential impacts on higher trophic levels of marine species (Corliss et al., 2024; Pérez‐Matus et al., 2025). Moreover, lowered productivity may result in less contribution of nearshore habitats to the global carbon cycle, especially in fjords, which are estimated to have the highest carbon sink per unit area on the planet (McHenry et al., 2025; Smith et al., 2015). Finally, without accurate historical baselines to measure from, ecologists and marine managers risk falling victim to shifting baseline syndrome (Pauly, 1995), in which the true effects of climate change and associated environmental degradation are underestimated due to recency bias. Our study exemplifies how quickly historical baselines may be lost, especially when physiological thresholds are reached during extreme events, resulting in the rapid local extinction of a species.

## AUTHOR CONTRIBUTIONS

Brian Timmer, Julia K. Baum, and Christopher J. Neufeld conceived of and secured funding for the study. All authors contributed to data collection and curation. Brian Timmer and Luba Y. Reshitnyk analyzed data with input from Julia K. Baum. Brian Timmer wrote the paper with input from all authors.

## FUNDING INFORMATION

Project funding came from Fisheries and Oceans Canada under the Aquatic Ecosystems Restoration Fund (AERF; 23‐AERF‐PAC‐036), as well as an Explorer Grant from The National Geographic Society and a Trebek Initiative grant from the Royal Canadian Geographical Society (019). B. Timmer was funded by an NSERC PGS‐D award.

## CONFLICT OF INTEREST STATEMENT

The authors declare no conflicts of interest.

## Supporting information


Appendix S1.

## Data Availability

Data and code (Timmer, 2026) are available in Zenodo at https://doi.org/10.5281/zenodo.18526498.
