# Supplementary material for: Historical data reveal extirpation of foundation species and kelp forest community deborealization in a coastal hotspot
Source: Ecol Appl. 2026 Apr 6;36(3):e70223. doi: 10.1002/eap.70223 (PMC13053217; doi:10.1002/eap.70223)
Supplement: Supplementary file 1 — Appendix S1. [file EAP-36-e70223-s001.pdf]

Appendix S1

Historical data reveal extirpation of foundation species and kelp forest community deborealization in a coastal hotspot

Brian Timmer, Luba Reshitnyk, Christopher J. Neufeld, Julia K. Baum

Ecological Applications

Supplemental Tables

Table S1. Sonde cast stations off Cape Lazo and Willemar Bluffs.

Station	Latitude	Longitude
1	49.68983	-124.85841
2	49.68992	-124.85476
3	49.67297	-124.88322
4	49.67062	-124.88152

Table S2. Landsat MSS imagery manually inspected for bull kelp presence/absence.

Sensor	Date
Landsat 01	1972-07-31
Landsat 01	1972-08-19
Landsat 01	1972-09-24
Landsat 01	1973-07-27
Landsat 01	1973-08-13
Landsat 01	1973-08-31
Landsat 01	1974-08-08
Landsat 01	1974-08-26
Landsat 01	1974-09-13
Landsat 02	1975-06-19
Landsat 02	1975-07-26
Landsat 02	1975-08-13
Landsat 02	1975-08-12
Landsat 01	1975-09-08
Landsat 01	1975-09-09
Landsat 02	1975-09-17
Landsat 02	1976-06-13
Landsat 01	1976-08-19
Landsat 01	1976-09-20
Landsat 03	1978-07-01
Landsat 02	1978-07-28
Landsat 03	1978-08-05
Landsat 02	1989-08-09
Landsat 02	1979-09-14
Landsat 02	1980-09-09
Landsat 02	1981-08-17
Landsat 03	1982-06-28

Table S3. Functional group allocation of species surveyed.

Species	Taxa
Desmerestia aculeata	Kelps
Desmerestia ligulata	Kelps
Alaria marginata	Kelps
Costaria costata	Kelps
Neoagarum fimbriatum	Kelps
Cymathaere triplicata	Kelps
Hedophyllum nigripes	Kelps
Nereocystis luetkeana	Kelps
Saccharina latissima	Kelps
Chondracanthus corymbifera	Red blades
Chondracanthus exasperatus	Red blades
Erythrophyllum oblongata	Red blades
Mazzaella splendens	Red blades
Mazzaella oregona	Red blades
Savoiea spp.	Red blades
Schizymenia pacifica	Red blades
Sparlingia pertusa	Red blades
Pyropia spp.	Red blades
Ceramium pacificum	Thick turfs
Callophyllis spp.	Thick turfs
Constantinea subulifera	Thick turfs
Cryptosiphonia woodii	Thick turfs
Devaleraea mollis	Thick turfs
Farlowia mollis	Thick turfs
Gracilaria spp.	Thick turfs
Grateloupia californica	Thick turfs
Leathesia difformis	Thick turfs
Mastocarpus spp.	Thick turfs
Mazzaella affinis	Thick turfs
Mazzaella rosea	Thick turfs
Microcladia coulterii	Thick turfs
Neogastroclonium subarticulatum	Thick turfs
Odonthalia floccosa	Thick turfs
Opuntiella californica	Thick turfs
Osmundea spectabilis	Thick turfs
Plocamium pacificum	Thick turfs
Polyneura latissima	Thick turfs
Prionitis spp.	Thick turfs
Sarcodiotheca gaudiichaudii	Thick turfs
Ulva intestinalis	Thick turfs
Ulva linza	Thick turfs
Herponema spp.	Thin turfs
Hymenena spp.	Thin turfs
Polyiphonia spp	Thin turfs
Symphyocladia spp.	Thin turfs
Ulva_sp	Green blades
Calliarthron tuberculosum	Articulate corallines
Corallina spp.	Articulate corallines
Zostara marina	Eelgrass
Lomentaria hakodatensis	Introduced
Mazzaella japonica	Introduced
Sargassum muticum	Introduced

Table S4. Environmental changes between 1972 and 2023 at the Chrome Island Lighthouse.

Season	SST	PSU
Annual	+1.27 °C; 95% CI: 0.84 to 1.69 °C	-1.04 PSU; 95% CI: -1.22 to -0.86 PSU
Summer	+1.66 °C; 95% CI: 1.20 to 2.13 °C	-0.74 PSU; 95% CI: -1.11 to -0.36 PSU
Winter	+0.65 °C; 95% CI: 0.46 to 0.84 °C	-1.82 PSU; 95% CI: -2.13 to -1.51 PSU

Table S5. SIMPER analysis output from 'Vegan' package in 'R' software, with species listed in order of highest to lowest contribution towards community dissimilarity between historical and modern timepoints. Here, the 'average' column refers to the species' contribution to the average between-group dissimilarity for both timepoints.

Species	average	sd	ratio	1972 average % cover	2023 average % cover	Cumulative contribution	P-value
Saccharina latissima	0.17171	0.20414	0.8411	29.26	6.073	0.189	0.005
Plocamium pacificum	0.11441	0.15448	0.7406	21.208	8.031	0.315	0.052
Mazzaella splendens	0.10237	0.14277	0.7171	21.094	0.323	0.427	0.001
Sargassum muticum	0.08598	0.15365	0.5596	3.51	10.677	0.522	0.647
Ulva spp.	0.06724	0.14037	0.479	4.01	8.562	0.596	0.846
Hedophyllum nigripes	0.05067	0.13274	0.3818	4.865	3.25	0.651	0.416
Chondracanthus corymbiferus	0.04965	0.09412	0.5276	0.583	7.521	0.706	0.011
Mazzaella japonica	0.04666	0.10622	0.4393	0	8.042	0.757	0.056
Prionitis spp.	0.04183	0.07423	0.5636	6.156	3.719	0.803	0.526
Neoagarum fimbriatum	0.02753	0.10865	0.2533	0	4.385	0.834	0.908
Lomentaria hakodatensis	0.02582	0.0563	0.4587	0.042	4.302	0.862	0.035
Sarcodiotheca gaudichaudii	0.01673	0.05154	0.3247	0.542	1.531	0.88	0.254
Costaria costata	0.01059	0.04343	0.2438	1.135	0.438	0.892	0.196
Chondracanthus exasperatus	0.00958	0.02995	0.3199	0.604	1.104	0.902	0.825
Desmerestia ligulata	0.00887	0.03028	0.293	0.062	1.219	0.912	0.555
Polyneura latissima	0.0084	0.03273	0.2566	2.188	0.083	0.921	0.011
Zostera marina	0.00799	0.05936	0.1345	0.073	0.792	0.93	0.659
Mastocarpus spp.	0.00689	0.02664	0.2585	1.521	0.073	0.938	0.007
Erythrophyllum oblangata	0.00557	0.02765	0.2014	1.521	0.042	0.944	0.003
Microcladia coulteriii	0.00518	0.02272	0.228	0.969	0.177	0.95	0.167
Osmundea spectabilus	0.00515	0.01616	0.3187	0.208	0.531	0.955	0.292
Alaria marginata	0.00436	0.0225	0.1938	0.354	0.333	0.96	0.672
Constantinea subulifera	0.00436	0.00986	0.4422	0.812	0.25	0.965	0.022
Ulva intestinalis	0.00356	0.02117	0.1683	0.021	0.417	0.969	0.761
Cymathaere triplicata	0.00342	0.0128	0.2674	0.531	0	0.973	0.026
Desmerestia aculeata	0.00245	0.00551	0.4446	0.365	0.01	0.975	0.001
Callophyllis spp.	0.00241	0.00418	0.5757	0.25	0.229	0.978	0.016
Mazzaella oregona	0.0022	0.00529	0.4163	0.312	0.115	0.98	0.139
Sparlingia pertusa	0.00166	0.00288	0.5773	0.271	0.094	0.982	0.127
Schizymenia pacifica	0.0016	0.00328	0.4887	0.042	0.229	0.984	0.008
Ceramium pacificum	0.00158	0.00333	0.4729	0.177	0.073	0.986	0.004
Gracileria sp.	0.00148	0.00524	0.2833	0.104	0.094	0.987	0.57
Hymenia sp,	0.00146	0.00309	0.4725	0.115	0.177	0.989	0.626
Grateloupia californica	0.00115	0.0038	0.3017	0.021	0.135	0.99	0.149

Mazzaella affinis	0.00112	0.00258	0.4364	0.01	0.156	0.991	0.002
Odonthalia flocossa	0.00096	0.00334	0.2859	0.115	0.062	0.993	0.123
Polysiphonia spp.	0.00095	0.00517	0.183	0	0.104	0.994	0.1
Cryptosiphonia woodii	0.00089	0.00314	0.2844	0.146	0	0.995	0.002
Savoia sp.	0.00068	0.00407	0.1674	0.073	0.01	0.995	0.019
Leathesia difformis	0.00063	0.00405	0.156	0	0.083	0.996	0.769
Herposiphonia plumosa	0.00053	0.00175	0.3059	0.062	0	0.997	0.034
Calliartharon tuberculosum	0.00044	0.00202	0.2188	0.021	0.031	0.997	0.844
Ulva linza	0.00042	0.00402	0.1045	0.01	0.021	0.998	0.088
Devaleraea mollis	0.0004	0.00112	0.3612	0.062	0	0.998	0.004
Pyropia spp.	0.00036	0.0022	0.1643	0	0.073	0.998	0.962
Corallina spp.	0.00035	0.0015	0.2331	0.01	0.031	0.999	0.336
Mazzaella rosea	0.00033	0.00286	0.1141	0.031	0	0.999	0.655
Neogastroclonium subarticulatum	0.00028	0.00129	0.2188	0.021	0.021	0.999	0.415
Nereocystis luetkeana	0.00024	0.00186	0.1285	0.021	0	1	0.676
Opuntiella Californica	0.00013	0.0007	0.1903	0	0.021	1	0.934
Farlowia mollis	0.00009	0.00048	0.1957	0.021	0	1	0.063
Symphocladia sp.	0.00008	0.00041	0.2003	0.021	0	1	0.039

Supplemental Figures

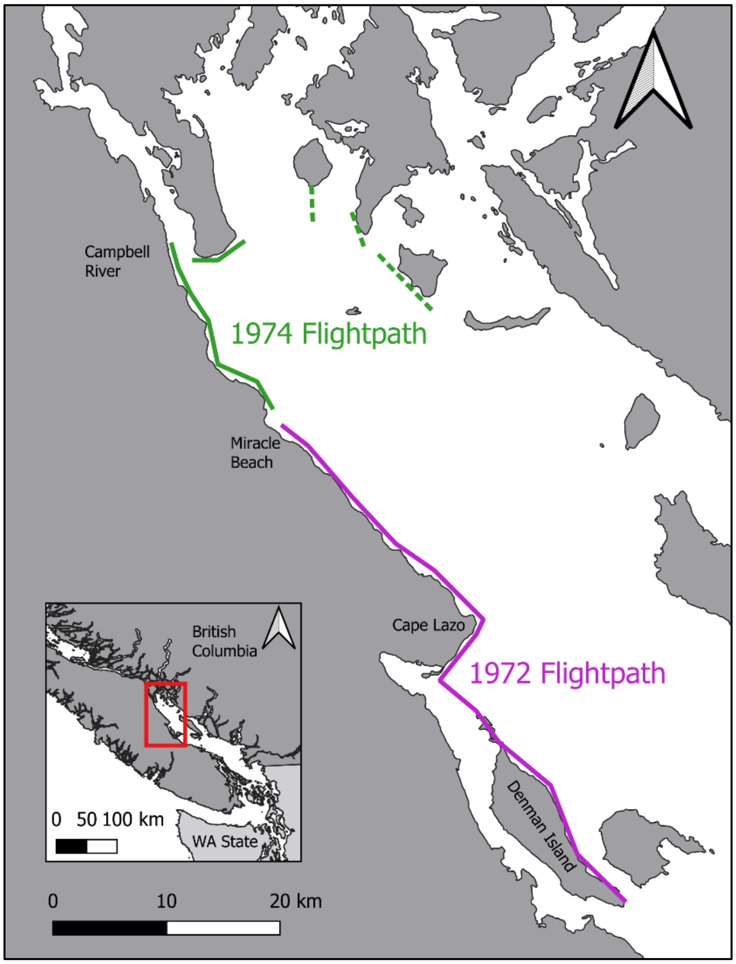

Figure S1. Map showing locations for collection of historical aerial photographs with both 1972 (purple) and 1974 (green) flightpaths highlighted. Solid lines for both 1972 and 1974 flightpaths were reflown by the Hakai Airborne Coastal Observatory in 2023. Dashed green lines on east side of the Strait of Georgia represent historical flightpaths that were not resurveyed.

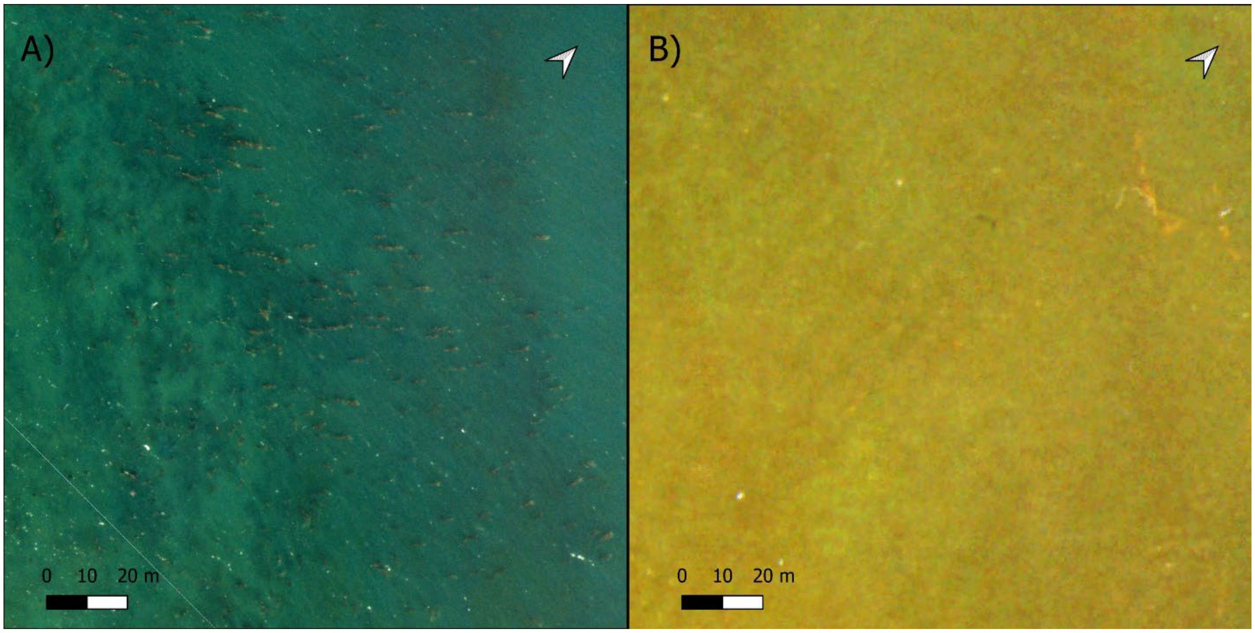

Figure S2. Example of differences between A) 1:2500 scale and B) 1:10000 scale aerial photographs for visual delineation of surface canopy kelp. Photographs clipped from overlapping geographical locations, georeferenced using immovable shoreline features.

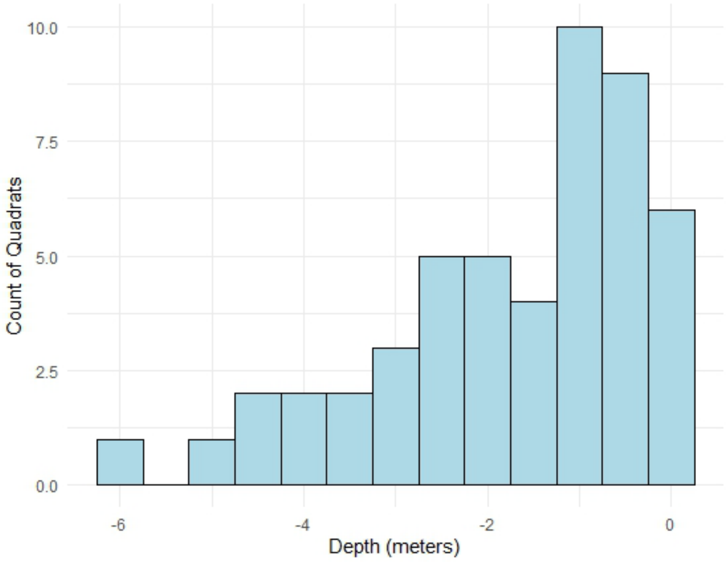

Figure S3. Histogram showing the bias towards shallower quadrats, due to the original historical surveys being used as ground truth surveys with a focus on shallow vegetation for detection in aerial imagery.

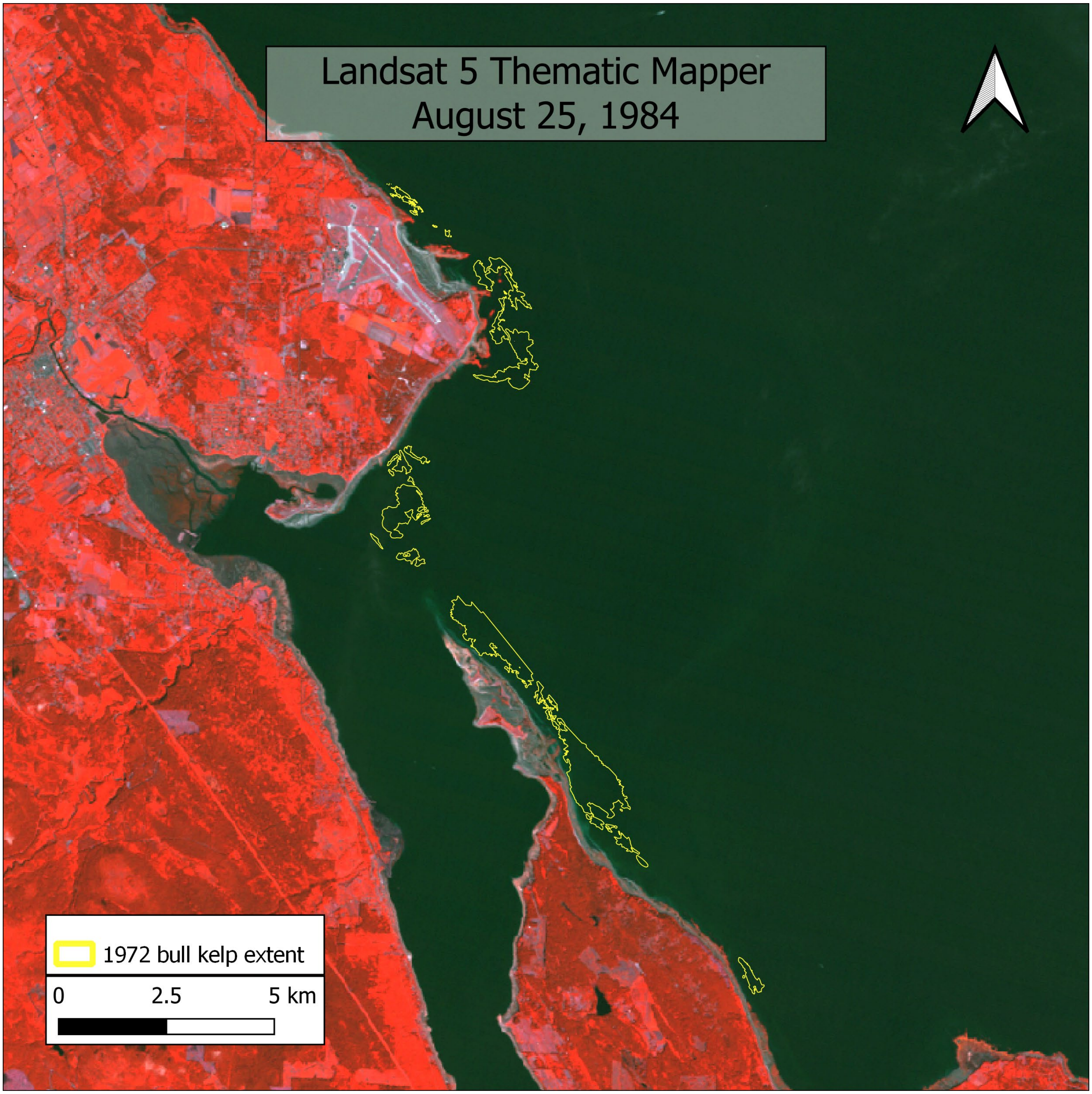

Figure S4. A false-color Landsat 5 image from 1984 with overlaid 1972 kelp distribution (yellow polygons) to demonstrate that no bull kelp surface-canopy is present in the imagery. The false color image replaces the red band with the near infrared band so that all vegetation glows red. The lack of red glow within the yellow polygons indicates that no vegetation is present at the surface.
